# Where Do Early Career Researchers Stand on Open Science Practices? A Survey Within the Max Planck Society

**DOI:** 10.3389/frma.2020.586992

**Published:** 2021-01-22

**Authors:** Daniel Toribio-Flórez, Lukas Anneser, Felipe Nathan deOliveira-Lopes, Martijn Pallandt, Isabell Tunn, Hendrik Windel, on behalf of Max Planck PhDnet Open Science Group

**Affiliations:** ^1^ Max Planck Institute for Research on Collective Goods, Bonn, Germany; ^2^ Max Planck Institute for Brain Research, Frankfurt Am Main, Germany; ^3^ Max Planck Institute for Plasma Physics, Munich, Germany; ^4^ Max Planck Institute for Biogeochemistry, Jena, Germany; ^5^ Max Planck Institute of Colloids and Interfaces, Potsdam, Germany; ^6^ Max Planck Institute for Physics, Munich, Germany

**Keywords:** survey, replication studies, registered report, preregistration, open data, open access, early career researchers, open science

## Abstract

Open science (OS) is of paramount importance for the improvement of science worldwide and across research fields. Recent years have witnessed a transition toward open and transparent scientific practices, but there is still a long way to go. Early career researchers (ECRs) are of crucial relevance in the process of steering toward the standardization of OS practices, as they will become the future decision makers of the institutional change that necessarily accompanies this transition. Thus, it is imperative to gain insight into where ECRs stand on OS practices. Under this premise, the Open Science group of the Max Planck PhDnet designed and conducted an online survey to assess the stance toward OS practices of doctoral candidates from the Max Planck Society. As one of the leading scientific institutions for basic research worldwide, the Max Planck Society provides a considerable population of researchers from multiple scientific fields, englobed into three sections: biomedical sciences, chemistry, physics and technology, and human and social sciences. From an approximate total population of 5,100 doctoral candidates affiliated with the Max Planck Society, the survey collected responses from 568 doctoral candidates. The survey assessed self-reported knowledge, attitudes, and implementation of different OS practices, namely, open access publications, open data, preregistrations, registered reports, and replication studies. ECRs seemed to hold a generally positive view toward these different practices and to be interested in learning more about them. Furthermore, we found that ECRs’ knowledge and positive attitudes predicted the extent to which they implemented these OS practices, although levels of implementation were rather low in the past. We observed differences and similarities between scientific sections. We discuss these differences in terms of need and feasibility to apply these OS practices in specific scientific fields, but additionally in relation to the incentive systems that shape scientific communities. Lastly, we discuss the implications that these results can have for the training and career advancement of ECRs, and ultimately, for the consolidation of OS practices.

## 1. Introduction

Open science (OS) is an umbrella term referring to multiple strategies, approaches, and practices regarding how research is conducted and shared. In recent years, there has been a trend toward the implementation of more of these practices, which correspond to two main objectives. The first objective is to improve the dissemination of knowledge by making the output of scientific research publicly available and free of charge (OECD, 2015). Some examples of steps taken in this direction are the Budapest Open Access Initiative^1^ in 2001, the “Berlin Declaration on Open Access to Knowledge in the Sciences and Humanities”^2^ in 2003, the Lyon Declaration^3^ in 2014, as well as the “cOAlition S^4^” and the “OA 2020”^5^ initiatives. Along these lines, research institutions themselves have also started to propose a transition toward publication systems based on free access to scientific knowledge (e.g., Max Planck Digital Library White Paper; Schimmer et al., 2015). The second goal of OS is to increase the transparency of research workflows and, in turn, to improve the reproducibility of scientific findings (Nosek et al., 2015; Munafó et al., 2017). Initiatives, such as the creation of the “Center of Open Science”^6^ in 2013, aim to provide guidelines toward more transparent and reproducible science. However, despite the momentum that OS has recently gained, there is still a long way to go in the standardization of these strategies and practices.

In this process of steering toward a more “open” science, early career researchers (ECRs) are of crucial relevance, as current actors and future decision makers of the institutional change that inevitably accompanies the movement supporting OS (Stürmer et al., 2017). This includes, for example, their role as future editors or reviewers in the steady transformation of the publication system, their role as instructors at their respective institutions regarding the integration of OS standards in the educational curricula of new researchers, or their role as principal investigators in the hiring process of these new researchers, where OS standards can become a key part in their assessment and consideration. Hence, it is imperative to gain insight about where ECRs stand on OS and its practices. Are ECRs knowledgeable about OS practices? What opinion do ECRs have about these practices? And more importantly, do ECRs implement them when conducting research?

To address these questions, the Open Science working group of the Max Planck PhDnet conducted a survey to assess the stance of doctoral candidates from the Max Planck Society toward different OS practices. The Max Planck Society is one of the leading scientific institutions for basic research in Europe and worldwide and, therefore, constitutes a significant academic population, with representation of a wide range of scientific disciplines. The present article provides a summary of the survey findings and discusses their implications regarding potential differences across research fields, training opportunities, and policies within science.

### 1.1. Why Open Science?

The dissemination and reception of research results is an integral part of the scientific process. One of the main goals of the OS movement is to foster access to knowledge independent of nationality, affiliation, scientific background, and wealth (OECD, 2015). However, it is important to emphasize that the free accessibility to research publications is just one aspect of OS. In fact, open access (OA) could be considered to be the last step of OS that aims to transparently share all aspects of knowledge as early in the scientific process as possible (Nielsen, 2012). Thus, in recent years, the OS movement has increasingly emphasized the importance of transparency in research workflows, including data collection and analysis to improve the reproducibility of scientific findings (Miguel et al., 2014; Nosek et al., 2015).

Conducting science within this framework is intended to alleviate several problems that have long been recognized. One of them is the low degree of reproducibility of published findings across scientific fields (Baker, 2016). For example, it has been suggested that increasing the transparency of research workflows through the public preregistration of studies can improve reproducibility by decreasing the occurrence of questionable research practices, such as HARKing (i.e., hypothesizing after results are known) and p-hacking (i.e., misuse of statistical analyses to find and report results that are statistically significant; Foster and Deardorff, 2017; Munafó, 2016; Powers and Hampton, 2019). Interestingly, there has been a consistent increase in the number of preregistered studies that were published between 2014 and 2018 (Hardwicke and Ioannidis, 2018), indicating that the value of these approaches becomes more and more appreciated.

Furthermore, science is often considered a collective good, which in most cases is publicly funded. As a consequence, it is progressively more common that a basic requirement for researchers to receive public funds is to make their results openly available (Schiltz, 2018). Along these lines, the expansion of the audience having access to scientific results increases the potential societal impact of research and enables a broader discussion about its goals (e.g., citizen science; Tennant et al., 2016) and its implications on related policies. Although it is hard to directly measure this kind of impact, OA work seems to be more widely disseminated when considering other metrics than traditional journal and citation-based metrics (i.e., *altmetrics*), such as number of mentions in social media, assignment to citation databases by different users, and general media coverage (Wang et al., 2015). An exemplary milestone to address the issue of accessibility was the release of the Budapest Open Access Initiative^7^ in 2001, which argued for making research data and publications freely available under the OA framework. As a measurable example, since 2004, the number of research articles published in a format consistent with OA has been growing faster than articles published in non-OA journals (Tennant et al., 2016), notwithstanding that, to date, roughly half of all recently published scientific articles are not openly accessible (Jubb et al., 2017; Mittermaier et al., 2018).

Another issue OS could tackle is the slow-paced nature of the scientific process. In the end, the transparent and accessible knowledge that OS tries to promote “is shared and developed through collaborative networks” (Vicente-Saez and Martinez-Fuentes, 2018). Thus, important large-scale research projects of interdisciplinary nature, such as the development of computational models for entire nervous systems (Markram, 2006; Sarma et al., 2018) or a swift understanding of a global pandemic, like the recent COVID-19 crisis, can benefit from easy accessibility to datasets and rigorous collaboration (Sohrabi et al., 2020; Xu et al., 2020).

### 1.2. Risks and Benefits of OS for ECRs

In the current OS framework, we believe that a focus on ECRs is necessary. As actors in the steering process toward more open and transparent science, the stance that ECRs hold is of crucial relevance. Furthermore, ECRs will typically be less entrenched in their methods and workflows than more senior scientists. Therefore, reaching out to them at this time and educating them on OS practices would likely have the largest impact. However, the benefits as well as the risks that OS might entail for ECRs could determine what ECRs think about OS and how they implement it. In what follows, we will review these potential benefits and risks.

Foremost among the many benefits of OS is the increased robustness and reliability of science when all steps are openly documented and data are openly available. This is especially the case in fields that struggle with reproducibility (Allen and Mehler, 2019). While this benefits science as a whole, it is certainly also a boon to the individual scientist, as open science can protect oneself against unreproducible studies to a certain degree. This is an aspect that grant agencies are thoughtfully taking into account (Allen and Mehler, 2019). From a different angle, the use of open data is an excellent opportunity for ECRs who often have neither the funding nor the network of a senior scientist. Even when they can freely ask other researchers for access to certain data, those requests are less often honored than when they come from more senior researchers (Magee et al., 2014). By sharing data, ECRs can set up collaborations with other researchers more easily, which in turn can lead to new research opportunities and coauthorships. These open sharing practices can potentially result in several citations, one for code, one for data, and one for the actual article (Gewin, 2016). Moreover, making the outcome of one’s research openly accessible can positively influence its impact. For example, sharing data and code can bring recognition and citations from replication studies or meta-analyses of published and unpublished data, whereas the citations of OA articles have been reported to be 18% higher than those of non-OA articles (Piwowar et al., 2018). Thus, while conducting OS may initially require more work in the short term, it can greatly benefit one’s career in the long term. Last but not least, ECRs might appreciate the positive impact that OS practices have for the public society more broadly. OS has the potential to foster the scientific education of the general public (Zuccala, 2010), while being of great value for NGOs, policy makers, and the innovation sector (European Commission, 2012), especially in countries with less resources (Tennant et al., 2016).

However, ECRs might also see risks associated with OS. While ECRs seem to a have a positive opinion on receiving open data, they are somewhat wary of supplying it, with the fear of being scooped as main reason (Nicholas et al., 2019). Despite the available tools to avoid this risk (e.g., time stamped DOIs), it is difficult to counter this fear. The damage that scooping entails for ECRs, generally with none or only a couple of publications, could be relatively higher than when it affects an established researcher with a long publication list. This might be especially critical for ECRs pursuing their PhD, as they often need a certain number of first authorship articles to be able to defend their dissertation. Other negative factors that can be crucial when considering OS are flexibility and time. Some OS practices (e.g., preregistrations) can reduce the flexibility of the researcher to look at new unexpected findings or tweak their methods halfway. Although these practices are intended to reduce the researcher’s degrees of freedom when performing confirmatory research (Nosek et al., 2018), ECRs might perceive them as an obstacle under the pressure of conducting innovative research. In addition, OS practices generally require more work and time. Preparing data or code for others to access requires considerably more time than its preparation for personal use. ECRs are often employed on short-term contracts or limited stipend funding periods and might not have the time to go through these steps. Even in the most privileged cases, these extra efforts may not be valued appropriately by a scientific community who assesses research based on journal impact metrics and number of publications (Moher et al., 2018). Despite the fact that ECRs might see the value of OS, they might fear that their supervisors may not allow it or do not see it as a valuable use of their time (Gewin, 2016). And this ties in with their greatest worry, namely, the impact that OS practices could have on their career (Schönbrodt, 2019). As long as scientists are being evaluated on traditional journal metrics, there are few incentives from a career perspective to fully commit to OS. If preregistrations or the publication of null findings are not valued or the DOIs for open code or data have no place in a scientist’s evaluation, then OS will struggle to find a strong place in science (Allen and Mehler, 2019).

### 1.3. Previous Work on Researchers’ Views on OS

Considering the previous, from the perspective of ECRs, OS can have positive and negative aspects. Previous work might help us to better understand the status quo of ECRs’ attitudes toward OS practices. To our knowledge, there is limited empirical evidence about which views researchers, and in particular ECRs, hold toward different OS practices.

In a longitudinal examination, Nicholas et al. (2019, 2020) surveyed 116 ECRs from seven different countries during a 3-year period. The survey assessed the participants’ views on OS in general, with special interest on scholarly communications. While many of the respondents showed poor understanding of OS practices, the study found a discrepancy in attitudes between respondents from different geographical locations. For instance, French ECRs viewed OS practices as a new form of unnecessary evaluation, while ECRs from China saw it as a form of taking back control over the scientific production pipeline. The study suggests a dual attitude toward OS. Some of the OS practices are seen as a social improvement (e.g., open access), whereas others are seen as potentially dangerous (e.g., open peer review). A cross-sectional extension of the previous study, including a much larger sample (1,051 respondents; Jamali et al., 2020), revealed that ECRs from different countries indeed differed in the usage of OA publishing and they also varied in their opinion about OA publications. However, ECRs generally tended to value the advantages of publishing in the OA format more than the disadvantages, especially the possibilities of increasing the visibility of their work and of reaching a broader audience (Jamali et al., 2020).

Using a different approach, Campbell et al. (2019) sent data-sharing requests to almost 500 corresponding authors of published articles on animal biotelemetry. The study showed a substantially higher response rate from corresponding authors who were ECRs than from those who were senior researchers. Campbell et al. (2019) argued that their findings were indicative of the positive predisposition toward data sharing and open data that ECRs have.

Another study concerning questionable practices in academia can further assist in better understanding the stance of ECRs regarding OS practices. Stürmer et al. (2017) surveyed 88 ECRs from the Social Psychology Section of the German Psychology Association. The aim of the survey was to assess the perceived prevalence of 14 different questionable research practices (e.g., data manipulation and HARKing), as well as the perceived causes of such behaviors. While the results indicate that most of the surveyed ECRs were less likely to believe serious frauds are committed in their academic environment, a good proportion believed that less severe misconducts are often practiced (e.g., to only report studies showing significant results). Crucially, OS practices were seen as a possible solution to some of these problems by most respondents. However, the sample size and the limited representativeness of the surveyed sample compromise the extrapolation of these results to the broader scientific community.

## 2. Research Overview

Under the premise that ECRs are of special importance in the transition toward OS, we conducted a survey intended to contribute to a further understanding of the stance of ECRs on OS. The survey assessed the knowledge, attitudes, perceived need, and implementation that ECRs report with regard to different OS practices, namely, OA publications, open data, preregistrations, registered reports, and replication studies. Furthermore, we analyzed the relationship between the reported knowledge, attitudes, perceived need, and implementation of these OS practices. Despite the correlational nature of the data and, therefore, the impossibility of drawing causal inferences, the examination of the relationship between these variables could provide useful insight regarding the estimation and forecasting of one variable (e.g., implementation of OS practices), given one of the others (e.g., knowledge about them). Prior to describing the conducted survey, we want to present some background information about the targeted population of ECRs and the Max Planck Society.

With more than 5,100 doctoral candidates, the Max Planck Society provided a convenient pool of ECRs, representing a broad diversity of international and disciplinary backgrounds. Although the Max Planck Society is based in Germany, roughly half of its affiliated doctoral candidates (47%) are expatriates, mainly from European and Asian countries but also from North and South America, Africa, and Australia (see MaxPlanck PhDnet Report 2018, Regler et al., 2019). The interdisciplinary nature of the research conducted in the 86 Max Planck Institutes, belonging to the Max Planck Society, hinders a clear-cut distinction between the research fields in which ECRs work. However, Max Planck Institutes are englobed into three broad sections. In the biomedical sciences (BM) section, different life sciences are represented including biology, medicine, ecology and their intersections. The chemistry, physics, and technology (CPT) section not only includes chemistry, physics, and applied technology but also mathematics and computer sciences. Finally, the human sciences (HUM) section collapses a broad range of disciplines within the humanities and social sciences from linguistics to economics, psychological and cognitive sciences, sociology, political sciences, history, law, art, and religious studies.

At the institutional level, the Max Planck Society has been actively involved in important OS initiatives, focused on fostering the open access of scientific publications (e.g., “OA2020 initiative”) and the implementation of OA publishing business models (see Max Planck Digital Library White Paper; Schimmer et al., 2015). However, one should not automatically assume that researchers within the Max Planck Society necessarily align with this institutional vision. The Max Planck Society is a strongly decentralized organization, in which each Max Planck Institute operates rather independently. Thus, the organizational culture and shared values that might shape ECRs’ attitudes and practices (in this case, regarding OS) is unlikely to offer a homogeneous picture. Instead, it is more plausible to expect heterogeneity in the attitudes and implementation of OS practices across institutes, which would resemble the differences between the research fields they represent. Although considering Max Planck Institutes as a unit of study would offer a fine-grained analysis of these differences, they substantially differ in terms of size (i.e., number of researchers), which would compromise the reliability and statistical power of these comparisons. Therefore, we will use the aforementioned sections (i.e., BM, CPT, and HUM) to examine whether the stance of ECRs toward the different OS practices vary across them.

Lastly, we would like to highlight that we, the authors of the present article, conducted this research as members of the Open Science working group of Max Planck PhDnet. Max Planck PhDnet is the organized network of all doctoral researchers affiliated with the Max Planck Society. Thus, we do not represent the opinion or interest of other members of the Max Planck Society. Within the Max Planck PhDnet, the Open Science working group aims to promote open and transparent scientific practices within the Max Planck Society. As members of this working group, we acknowledge our own positive disposition toward OS. This is the reason why we took special care to keep the survey and our analysis objective both to adhere to good scientific practices and to avoid biased interpretations of crucial data for our own goal of promoting OS. Thus, all material and anonymized data have been made available for further reference.

## 3. Methodology

### 3.1. Survey Construction

The online survey was set up in LimeSurvey and hosted by the Max Planck Society (https://umfragen.vw.mpg.de/). The survey was active from June 19th to July 22nd, 2019. Doctoral researchers affiliated with a Max Planck Institute received an invitation with a personalized link to the survey via email. Due to the decentralization of the Max Planck Society, the access to a complete and updated list of email addresses was difficult. On the date of activation of the survey, we had access to 3,498 email addresses from doctoral researchers. With the purpose of including doctoral researchers that were not in this list (e.g., newly arrived), we further shared the survey via an open link with the representatives of doctoral researchers of every Max Planck Institute and asked them to forward it to their peers. At the beginning of the survey, participants gave informed consent, which introduced the objective of the survey and stated that participation was voluntary and completely anonymous. Participants also consented to the publication of the anonymized, aggregated data in scientific journals endorsing open access standards. Any incomplete response of the survey was strictly excluded from analysis.

The first part of the survey introduced a battery of questions for each of the different OS practices. The questions of a respective OS practice were presented in an independent page, and the different pages were sequentially presented in the following order: OA publications, open data, preregistrations, registered reports, and replication studies. In each page, a definition of the respective OS practice was given before introducing any question (see Table 1). Then, we presented questions assessing ECRs’ knowledge, attitudes, perceived need, and implementation of the respective OS practice. In a second part, the survey collected information about the support that ECRs received with regard to OS by their institutes. This part is not reported in the current article, as it was only intended for internal purposes and extends the goal of the present work. The last part of the survey included questions about demographic information, such as gender, age, and scientific section. In total, the survey contained 51 unconditional questions and six conditional questions. For every question in the survey, we enabled the answer option "No answer" or "I do not want to answer this question" in order to allow participants to decide on a voluntary basis what information they wanted to provide. Moreover, the obtained data were saved according to the German data protection guidelines. A complete version of the survey is accessible in the Supplementary Material.

**TABLE 1:** Definition of OS practices included in the survey.

**OS Practice**	**Definition**
Open access publications	Unrestricted public availability of a research article on the Internet through formal OA publication systems (e.g., gold or hybrid OA publishers) or author self-archiving (e.g., working papers or preprints in online repositories).
Open data	Unrestricted public availability of research data and/or any resource necessary for the collection of these data (methodology, protocol, software packages, etc.) generally through online repositories.
Preregistrations	Detailed registration of hypotheses, study design, methodology, and analyses prior to data collection to which the researcher commits in order to guarantee a transparent confirmatory (vs. exploratory) test of the formulated hypotheses.
Registered reports	Publication format consisting of two peer-reviewed stages: 1) an in-principle acceptance of a research paper prior to data collection based on a detailed preregistration and 2) the actual publication of the research paper after data collection, including any result (statistically significant or not) obtained by following what was specified in the preregistration.
Replication studies	Studies intended to 1) reproduce a scientific finding from a previous study by recreating the critical elements that are assumed to explain the original result (i.e., close replication) or 2) generalize a scientific finding by purposefully modifying at least one component of the original study, such as the sample, the protocol, or the material used (i.e., conceptual replication).

### 3.2. Participants

The survey was completed by 568 doctoral researchers. This was 11% of the total population of doctoral researchers of the Max Planck Society, with a representation of 71 out of the 86 Max Planck Institutes. The average respondents’ age was 28 years. Overall, 50.4% of the respondents identified as male, 43.8% as female, 0.2% identified as other, and 5.6% did not answer this question. From the total number of respondents, 519 used the personalized link (15% response rate) and 49 used the open survey version. Across the three different scientific sections of the MPS, the respondents were distributed as follows: 36% from BM, 43% from CPT, 16% from HUM, and 5% did not provide this information. In the Max Planck Society, these three scientific sections are distinct in size (see MaxPlanck PhDnet Report 2018, Regler et al., 2019, p. 7). Therefore, the response rate from each section was normalized based on the total number of ECRs per section. According to this, the highest response rate relative to the total number of ECRs in each section was from HUM, specifically, 16.9% higher than the expected rate. CPT also showed an 8.6% response rate higher than the expected, while in BM, the relative response rate was 1.7% lower than the expected rate.

### 3.3. Measures

#### 3.3.1. Knowledge

ECRs reported the extent to which they were knowledgeable about the respective OS practice (henceforth, *current knowledge*) and the extent to which they would like to know more about that OS practice (henceforth, *desired knowledge*). The response scales ranged from 1 (not at all) to 7 (extremely). For most OS practices, these two measures did not significantly correlate. However, we observed them weakly correlated for OA publications—in this case, negatively, *r* = −0.12, *t* (566) = −2.768, *p* = 0.006, 95% CI [−0.20, −0.03]—and for registered reports—in this case, positively, *r* = 0.11, *t* (550) = 2.595, *p* = 0.010, 95% CI [0.03, 0.19]. We additionally computed a difference score to examine the gap between the desired and the current knowledge that ECRs reported for each OS practice (henceforth, *knowledge gap*), which ranged from −6 (i.e., high current knowledge and low desired knowledge) to 6 (i.e., low current knowledge and high desired knowledge).

#### 3.3.2. Attitude Valence

We measured ECRs’ attitudes toward each OS practice through bivariate semantic scales. ECRs reported the extent to which they found each OS practice *useful*, *useless*, *advantageous*, *disadvantageous*, *beneficial*, and *harmful*, using Likert scales from 1 (not at all) to 7 (extremely). Furthermore, we asked participants to report their attitudes three times, while considering one of the following perspectives: the perspective of their *daily research life*, the perspective of their *research field*, and the perspective of the *public society*. For each of these perspectives, we computed a difference score with each couple of antonym adjectives by subtracting the negatively valenced (e.g., useless) from the positively valenced (e.g., useful). Across OS practices and perspectives, these different scores showed high internal consistency (Cronbach’s *αs* ≥ 0.88). Therefore, we averaged the difference scores of the three pairs of adjectives and computed an overall index of attitude valence, with positive values (i.e., 0 to 6) representing an overall positive attitude and negative values (i.e., −6 to 0) representing an overall negative attitude.

#### 3.3.3. Perceived Need

Similarly, to our measure of attitudes, ECRs reported how *necessary* and *unnecessary* they found each OS practice, using Likert scales from 1 (not at all) to 7 (extremely) and considering the same perspectives (i.e., daily research life, research field, and public society). We also computed a difference index of perceived need, subtracting the score of the item “unnecessary” from the score of the item “necessary.” Therefore, positive scores (i.e., 0 to 6) indicate perceived need and negative scores (i.e., −6 to 0) indicate perceived lack of need.

#### 3.3.4. Implementation

ECRs reported whether or not they had implemented the respective OS practice in the past 12 months (henceforth, *past implementation*) and whether or not they planned to implement it in the following 12 months (henceforth, *future implementation*). The answers to these questions were coded 0 (no) and 1 (yes). In the case of OA publications, we further asked ECRs to specify where they had published/planned to publish their research article, with three response options (open access publisher, self-archiving, and other).

### 3.4. Statistical Analyses

To analyze the data, we mainly used linear and logistic mixed regression models to account for the dependency between participants’ repeated measures (e.g., across OS practices and perspectives). For this purpose, every tested model included the respondents’ anonymized IDs as a random factor.

A first set of analyses aimed to compare mean levels of knowledge, attitudes, perceived need, and implementation across OS practices and scientific sections. Therefore, OS practices, the ECRs’ scientific section, and the interaction between these two were included as fixed factors into our models. In the case of attitudes and perceived need, we additionally introduced the different perspectives ECRs considered (i.e., daily research life, research field, and public society) as an additional fixed factor to test whether differences emerged across them. When statistical models were parsimonious, we included and controlled for further demographic variables (i.e., age and gender). When interaction effects were significant, we performed pairwise comparisons to elucidate the differences between marginal means (e.g., between OS practices or scientific sections). In order to minimize the impact of multiple testing on our statistical inferences, the reported *p*-values of these comparisons were corrected using Bonferroni adjustments.

In a second set of analyses, we examined the relationship between the different measures of interest and whether this relationship varied across OS practices. When studying any relationship involving attitudes or perceived need, we also analyzed whether the considered perspectives affected the strength of the association between the investigated measures.

The evaluation of the data and statistical analysis were performed in R (R Core Team, 2019, version 3.6.2), using the packages *lme4* (Bates et al., 2015, version 1.1.21) and *lmerTest* (Kuznetsova et al., 2017, version 3.1.1). The complete anonymized dataset, together with full code, used to analyze the data is accessible at https://osf.io/qbgct/.

## 4. Results

### 4.1. Current vs. Desired Knowledge About OS Practices

**FIGURE 1:**
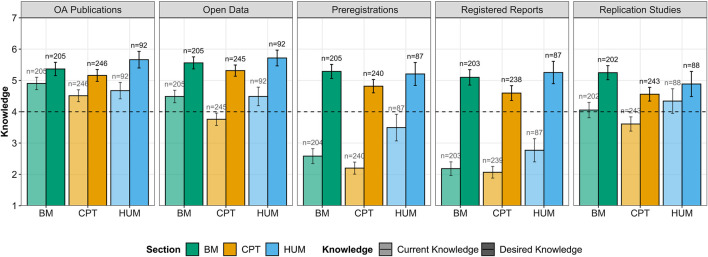
Mean of current vs. desired knowledge about different OS practices in the different sections (BM: biology and medicine, CPT: chemistry, physics, and technology, and HUM: humanities). Dashed line represents the midpoint of the scale (i.e., 4). Error bars represent 95% confidence intervals (CIs).

Figure 1 represents the observed mean levels of current and desired knowledge across OS practices and scientific sections.

ECRs showed a higher current knowledge about OA publications, open data, and replication studies than about preregistrations and registered reports. However, we observed a relative variation between sections for each OS practice, as indicated by the significant interaction between OS practices and section, *F* (8,2128) = 5.825, *p* < 0.001, ${\text{η}}_{p}^{2}$ = 0.020. Perhaps the most salient differences referred to preregistrations and registered reports. For these two OS practices, ECRs from the HUM section reported significantly higher current knowledge than those from the BM and CPT sections (*p*s ≤ 0.020). Furthermore, we observed significant differences suggesting that ECRs from CPT reported themselves less knowledgeable about open data (*p*s < 0.001) and replication studies (*p*s ≤ 0.010) than those from BM and HUM.

The levels of desired knowledge were always higher than levels of current knowledge. However, we also observed differences between sections as a function of the specific OS practice, as shown by a significant interaction effect, *F* (8,2129) = 2.567, *p* = 0.009, ${\text{η}}_{p}^{2}$ = 0.009. In general, ECRs from BM and HUM reported similar desired knowledge toward the different OS practices, whereas those from CPT reported significantly lower desired knowledge when compared to the BM and HUM sections for every OS practice (*p*s ≤ 0.032), except for open data where there were no significant differences between sections (*p*s ≥ 0.127).

We obtained additional information by examining the gap between current vs. desired knowledge. Once more, we observed a significant interaction, *F* (8,2130) = 5.732, *p* < 0.001, ${\text{η}}_{p}^{2}$ = 0.020, reflecting the variation of relative differences between sections across OS practices. More specifically, the knowledge gap did not significantly differ between sections with regard to OA publications, open data, registered reports, and replication studies, but it did so in the case of preregistrations. Specifically, BM and CPT did not differ from each other (*p* = 0.99), but HUM showed a significantly lower knowledge gap when compared to BM and CPT (*p*s ≤ 0.005).

Finally, when we included age and gender in our model, our results regarding knowledge gap did not change. However, while we did not register an effect of age, *F* (1,477) = 0.018, *p* = 0.893, ${\text{η}}_{p}^{2}$ = 0.000, we observed that gender significantly affected knowledge gap, *F* (1,480) = 8.187, *p* = 0.004, ${\text{η}}_{p}^{2}$ = 0.004. This gender effect indicated that female ECRs reported a larger gap between their desired and their current knowledge (*M* = 1.84) than men (*M* = 1.32). When we considered this gender effect independently for the measures of current and desired knowledge, the gender differences only reproduced with desired knowledge, *F* (1,479) = 5.382, *p* = 0.021, ${\text{η}}_{p}^{2}$ = 0.003. This indicated that women reported higher desired knowledge than men but similar current knowledge. The interaction between section and OS practices discussed above was not significant in this case, which suggests that the differences between sections on desired knowledge could be partly explained by gender differences.

### 4.2. Attitudes Toward OS Practices

**FIGURE 2:**
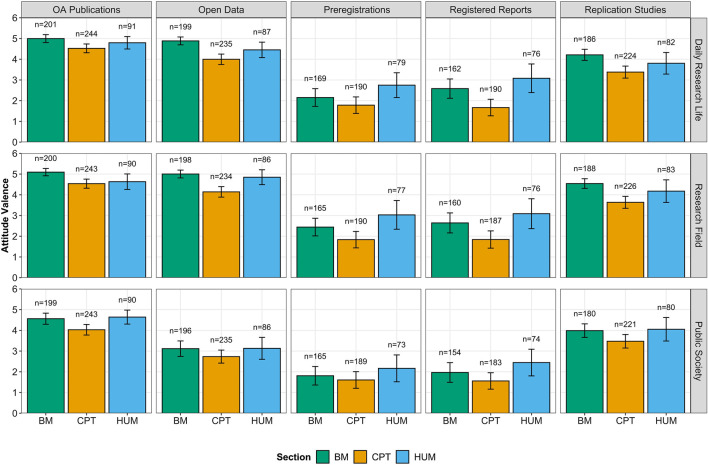
Attitude valence of ECRs toward OS practices from different perspectives (i.e., daily research life, research field, and public society) aggregated by sections (BM: biology and medicine, CPT: chemistry, physics, and technology, and HUM: Humanities). Error bars represent 95% confidence intervals (CIs).

Descriptively, we observed that ECRs held fairly positive attitudes regarding all OS practices across perspectives (see Figure 2). Attitudes were more saliently positive toward OA publications and open data, followed by replication studies, and they were less positive toward preregistrations and registered reports. Furthermore, the attitudes that ECRs reported were generally less positive when considering the perspective of the public society.

Our analyses provide a more nuanced picture of the effects of the OS practices, the considered perspective, and also the scientific sections. A significant interaction between OS practice and perspective indicated that those differences in the ECRs’ attitudes associated to the considered perspective (i.e., daily research life, research field, or public society) varied as a function of the specific OS practice, *F* (8,6614) = 12.707, *p* ≤ 0.001, ${\text{η}}_{p}^{2}$ = 0.015. With regard to OA publications (*p*s ≤ 0.020), open data (*p*s ≤ 0.001), preregistrations (*p*s ≤ 0.020), and registered reports (*p*s ≤ 0.003), ECRs held more positive attitudes regarding their daily research life and their research field in comparison to the public society. However, attitudes toward replication studies were more positive when it came to ECRs’ research field compared to their daily research life and the public society, although these differences did not reach statistical significance (*p*s ≥ 0.051). Furthermore, a second interaction between OS practice and sections, *F* (8,6669) = 4.079, *p* ≤ 0.001, ${\text{η}}_{p}^{2}$ = 0.005, showed that differences between sections varied across OS practices but with a general trend. That is, for every OS practice, ECRs from CPT generally held significantly less positive attitudes compared to ECRs from either BM or HUM (*p*s ≤ 0.007), while these two sections did not significantly differ between each other (*p*s ≥ 0.180).

Lastly, when we introduced age and gender into the model, some gender differences appeared concerning attitudes. In particular, a significant interaction between gender and OS practices, *F* (4,5900) = 11.352, *p* ≤ 0.001, ${\text{η}}_{p}^{2}$ = 0.008, indicated that while male and female ECRs held similarly positive attitudes regarding OA publications (*p* = 0.686), open data (*p* = 0.689), and replication studies (*p* = 0.506), women reported significantly more positive attitudes toward preregistrations (*p* = 0.027) and registered reports (*p* ≤ 0.001). Gender also interacted with the perspectives that ECRs considered to report their attitudes, *F* (2,5860) = 13.547, *p* ≤ 0.001, ${\text{η}}_{p}^{2}$ = 0.005. Female researchers reported more positive attitudes across OS practices when it related to their daily research life (*p* = 0.012) and their research field (*p* = 0.007) than male researchers. These gender differences did not appear when ECRs considered the public society perspective, with ECRs holding similarly positive attitudes, regardless of their gender (*p* = 0.336).

### 4.3. Perceived Need of OS Practices

**FIGURE 3:**
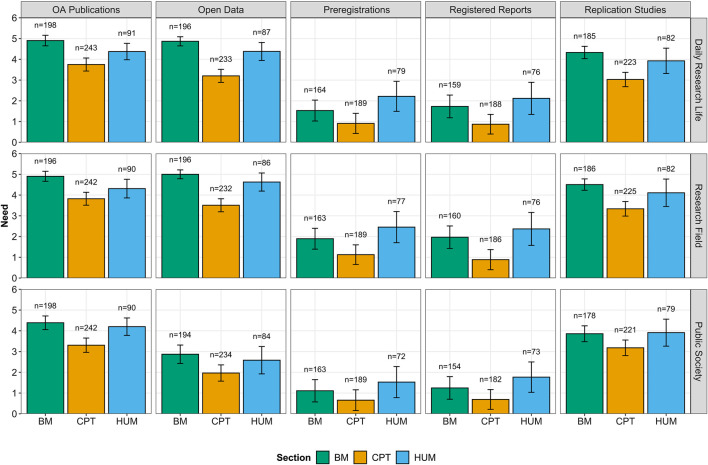
Perceived need of ECRs regarding OS practices from different perspectives (i.e., daily research life, research field, and public society) aggregated by sections (BM: biology and medicine, CPT: chemistry, physics, and technology, and HUM: humanities). Error bars represent 95% confidence intervals (CIs).

As described in Figure 3, ECRs perceived every OS practice at least moderately necessary, although there were important differences between OS practices, sections, and considered perspectives. From our tested model, we observed a significant interaction effect between the considered perspectives and the different OS practices, *F* (8,6560) = 11.430, *p* ≤ 0.001, ${\text{η}}_{p}^{2}$ = 0.014. This interaction depicted a general tendency to evaluate most OS practices as less necessary from the perspective of the public society than from the perspectives of the researchers’ daily research life and/or their research field. However, this difference did not emerge regarding replication studies, in which perceived need was similar from every perspective (*p*s ≥ 0.104). A second significant interaction between the different sections and the OS practices, *F* (8,6618) = 4.800, *p* ≤ 0.001, ${\text{η}}_{p}^{2}$ = 0.006, indicated once again that differences between sections varied across the different OS practices. Indeed, differences varied in size between sections, but the same pattern repeated across the different OS practices: ECRs from BM and HUM perceived every practice as significantly more necessary than those from CPT (*p*s ≤ 0.052).

Finally, when controlling for age and gender, we found gender differences in how ECRs perceived the need of the OS practices. According to the significant interaction between gender and OS practices, *F* (4,5849) = 11.590, *p* < 0.001, ${\text{η}}_{p}^{2}$ = 0.008, the need that male and female researchers perceived significantly differed regarding preregistrations (*p* = 0.033) and registered reports (*p* < 0.001) but not regarding OA publications (*p* = 0.613), open data (*p* = 0.791), and replication studies (*p* = 0.828). Specifically, female researchers reported higher need of the two firstly mentioned OS practices than men. Despite the interaction between gender, perspectives, and sections not being significant, *F* (4,5809) = 2.154, *p* = 0.072, ${\text{η}}_{p}^{2}$ = 0.001, there were some significant gender differences within the CPT section, where women reported higher levels of need of the studied OS practices in comparison to men, in particular regarding their daily research life (*p* = 0.009) and their research field (*p* = 0.005) but not the public society (*p* = 0.921). We also observed a similar tendency of women from the HUM section but only regarding the perspective of their research field (*p* = 0.042).

### 4.4. Implementation of OS Practices

**FIGURE 4:**
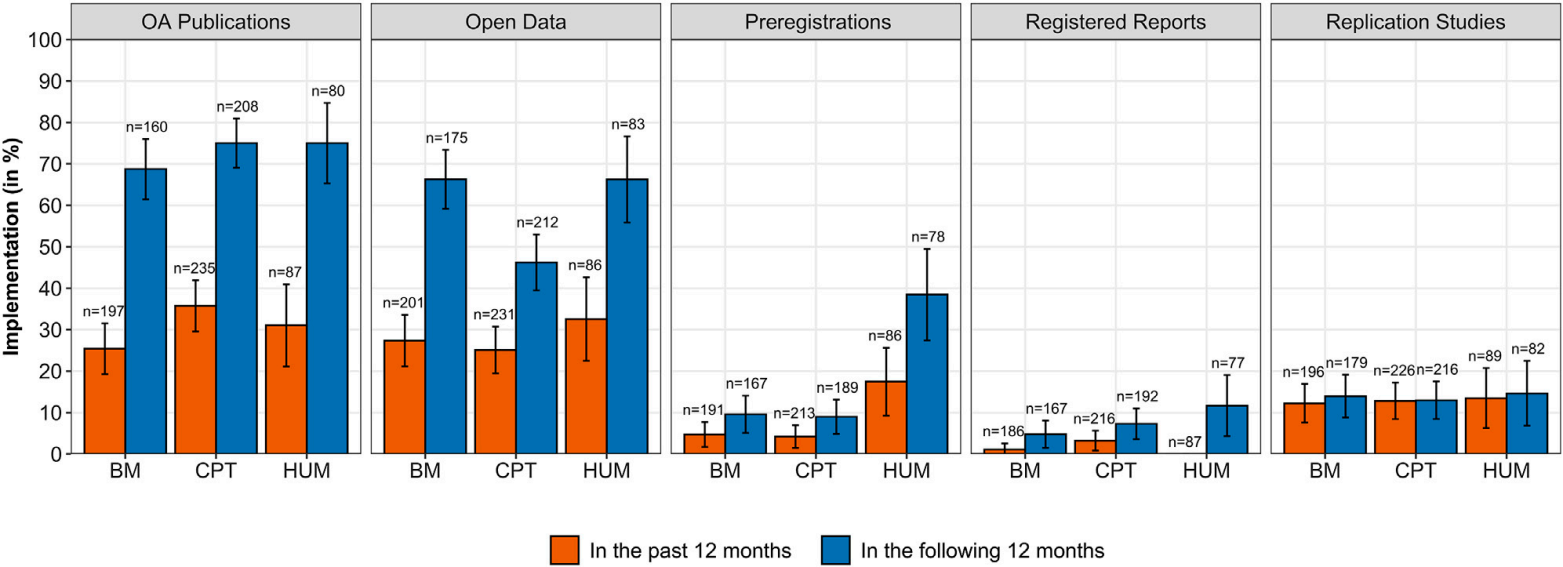
Frequencies of implementation of OS practices among ECRs in the past 12 months and planned implementation in the following 12 months across the different sections (BM: biology and medicine, CPT: chemistry, physics, and technology, and HUM: humanities). Error bars represent 95% confidence intervals (CIs).

In the case of implementation, we used a logistic mixed model to examine the interaction between OS practices and scientific sections. However, including this interaction term led to model convergence problems. Therefore, we decided to independently check in simple logistic regression models whether there were differences between sections in the use of each OS practice. The obtained results are summarized in Figure 4.

For OA publications, 31% of ECRs published at least one research article OA in the previous 12 months (25% in BM, 36% in CPT, and 31% in HUM). ECRs from CPT were significantly more likely to have published an OA publication than those from BM, OR = 1.64, 95% CI = [1.08, 2.49], while the ones from HUM did not significantly differ from the other two sections. Among the ECRs who answered that they published OA in the last 12 months, 64% used a formal OA publisher (80% in BM, 54% in CPT, and 63% in HUM), whereas 18% used self-archiving (6% in BM, 25% in CPT, and 19% in HUM) for making their preprints or working papers openly available. The remaining 18% either used other means or did not specify. The obtained data draw a more optimistic picture concerning ECRs’ willingness to publish OA in the future, with 73% of ECRs planning to publish OA in the following 12 months (69% in BM, 75% in CPT, and 75% in HUM). In this case, we did not observe significant differences across sections. From those who were willing to publish OA, 61% would do so via OA publishers (66% in BM, 56% in CPT, and 62% in HUM), 18% via self-archive repositories (14% in BM, 20% in CPT, and 23% in HUM), and the remaining 20% either planned to use other means or did not specify.

Similar to OA publications, 28% of ECRs made their research data openly available in the past 12 months, with small differences between sections (27% in BM, 25% in CPT, and 33% in HUM), which were not statistically significant. However, the picture slightly changed when it came to future data sharing. From the whole sample, 57% of ECRs planned to share their research data in the future but with some remarkable differences between sections. Specifically, ECRs from BM (66%, OR = 2.29, 95% CI [1.52, 3.47]) and HUM (66%, OR = 2.28, 95% CI [1.36, 3.92]) were significantly more likely to plan sharing their research data in the future than ECRs from CPT (46%).

Preregistrations had been used by 7% of ECRs in the past 12 months, but there were important differences between sections. While 17% of ECRs in the HUM section used preregistrations, in BM and CPT pre-registrations were significantly less likely − respectively, 5%, OR = 0.23, 95% CI [0.09, 0.55], and 4%, OR = 0.21, 95% CI [0.08, 0.49]. We observed similar but more pronounced differences in the willingness to implement preregistrations in the future for ECRs from HUM (38%) compared with ECRs from BM (10%, OR = 0.17, 95% CI [0.08, 0.33]) and CPT (9%, OR = 0.16, 95% CI [0.08, 0.31]).

As for replication studies, ECRs were similarly likely to participate or conduct this kind of studies in the past and in the following 12 months, with average levels ranging from 12 to 15% and no significant differences between sections. Registered reports showed the lowest frequencies, with a 2% of ECRs implementing this practice in the past 12 months and a relatively higher 7% in the future 12 months. We did not observe significant differences between sections neither in past nor in future implementation.

### 4.5. Relationship Between Knowledge, Attitudes, Perceived Need, and Implementation

#### 4.5.1. Knowledge and Attitudes

**FIGURE 5:**
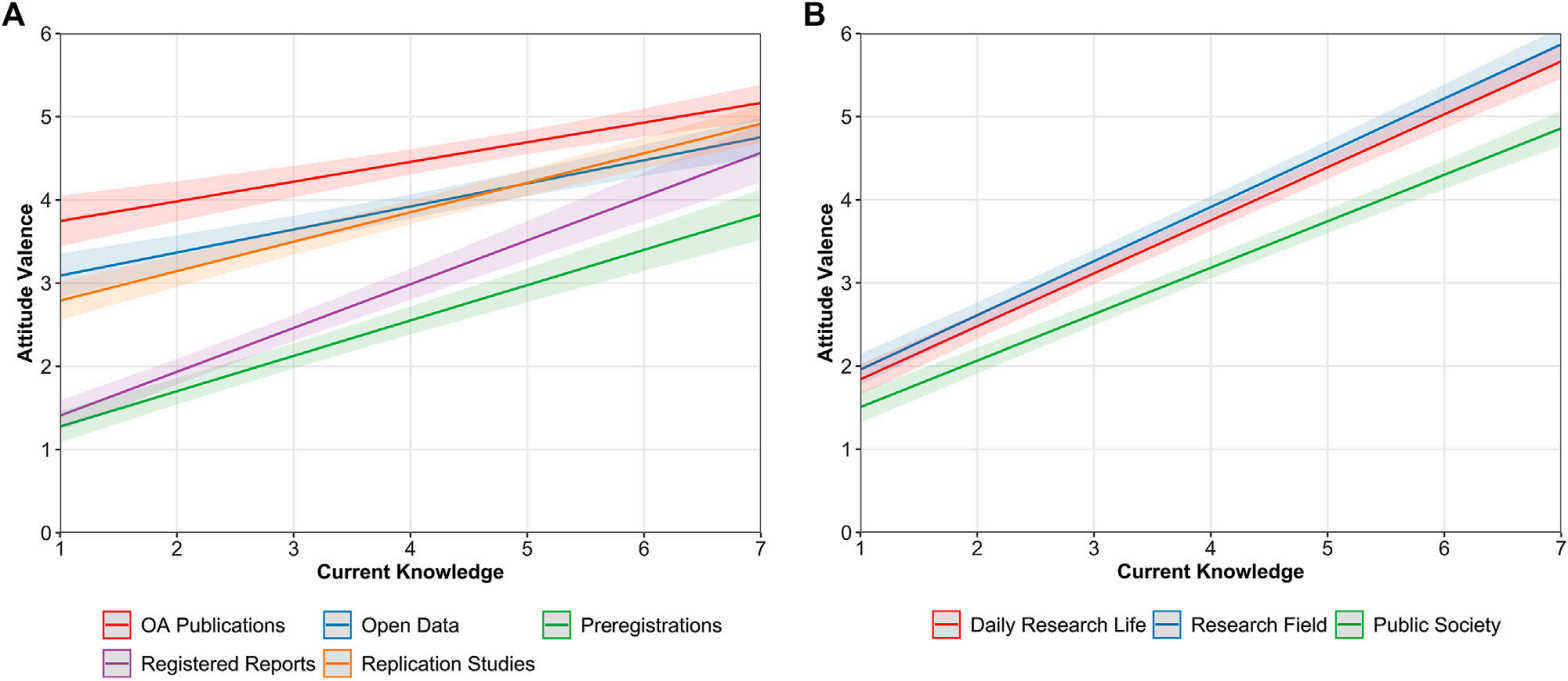
Predicted values of attitude valence as a function of ECRs’ current knowledge across OS practices **(A)** and different perspectives **(B)**. Bandwidths represent 95% confidence intervals (CIs).

We first analyzed whether the attitudes that ECRs held toward the different OS practices varied as a function of ECRs’ level of current and desired knowledge. We observed a positive relationship between holding positive attitudes and current knowledge, which indicated that ECRs who reported to have higher knowledge tended to hold more positive attitudes toward the different OS practices. However, the strength of this relationship differed across OS practices, as indicated by a significant interaction between knowledge and OS practices, *F* (4, 7226) = 11.075, *p* < 0.001, ${\text{η}}_{p}^{2}$ = 0.006 (see Figure 5A). Specifically, the association with ECRs’ attitude valence was significantly stronger for registered reports, *β* = 0.37, 95% CI [0.32, 0.42], followed by preregistrations, *β* = 0.30, 95% CI [0.26, 0.35], replication studies, *β* = 0.25, 95% CI [0.21, 0.29], open data, *β* = 0.20, 95% CI [0.15, 0.25], and lastly, OA Publications, *β* = 0.17, 95% CI [0.12, 0.22]. The relationship between attitudes and current knowledge was also significantly influenced by the perspectives that ECRs were asked to consider when reporting their attitudes, *F* (2, 4,982) = 10.128, *p* < 0.001, ${\text{η}}_{p}^{2}$ = 0.003 (see Figure 5B). For the ECRs’ daily research life, *β* = 0.45, 95% CI [0.42, 0.49] and research field, *β* = 0.46, 95% CI [0.43, 0.50], the association between attitudes and current knowledge was significantly stronger than for the public society, *β* = 0.40, 95% CI [0.36, 0.43].

**FIGURE 6:**
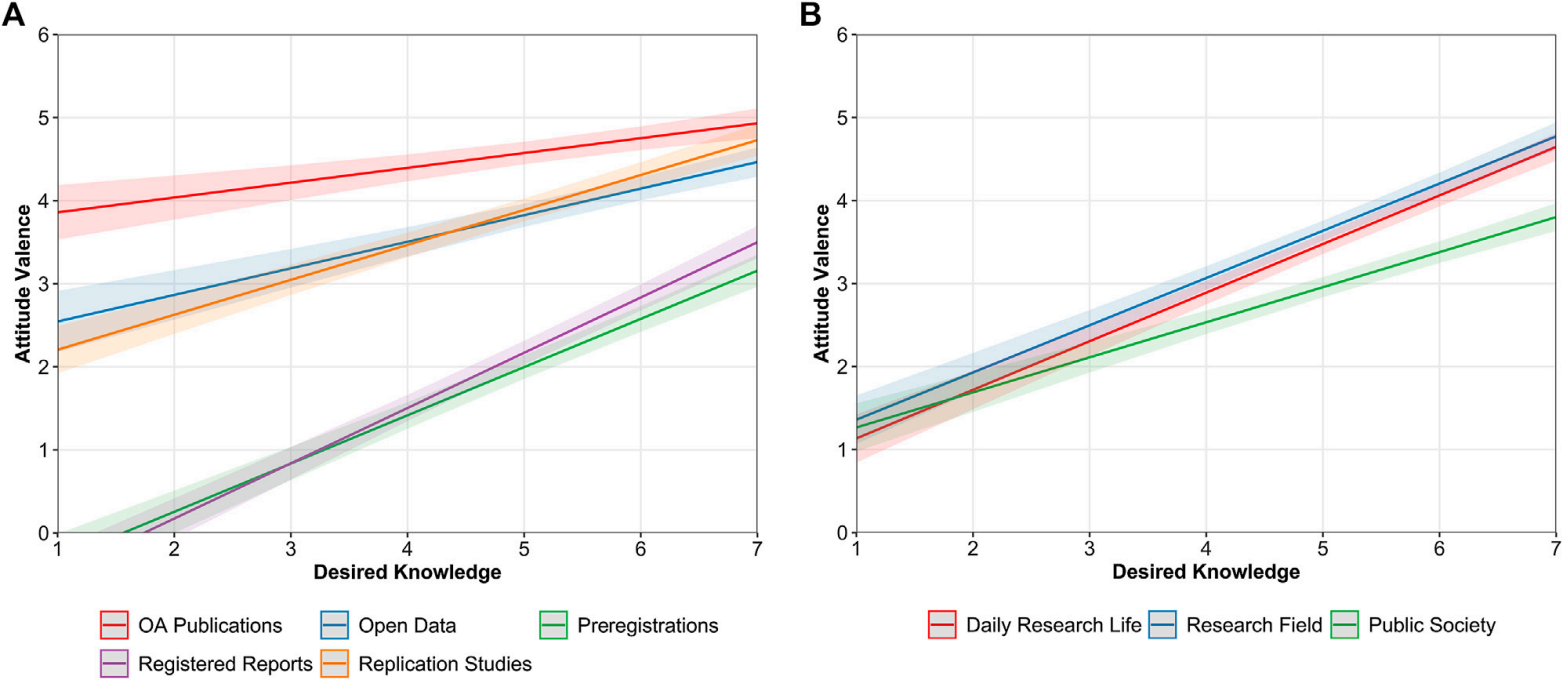
Predicted values of attitude valence as a function of ECRs’ desired knowledge across OS practices **(A)** and different perspectives **(B)**. Bandwidths represent 95% confidence intervals (CIs).

Similar differences emerged when considering levels of desired knowledge. Overall, there was a positive relationship between holding positive attitudes and the interest in acquiring more knowledge about the different OS practices, but it significantly differed across OS practices, *F* (4, 7195) = 35.456, *p* < 0.001, ${\text{η}}_{p}^{2}$ = 0.020 (see Figure 6A). This relationship was significantly stronger for registered reports, *β* = 0.41, 95% CI [0.37, 0.46], followed by preregistrations, *β* = 0.36, 95% CI [0.32, 0.40], replication studies, *β* = 0.26, 95% CI [0.22, 0.30], open data, *β* = 0.20, 95% CI [0.15, 0.25], and finally, OA publications, *β* = 0.11, 95% CI [0.07, 0.15]. It is noteworthy that with desired knowledge, intercepts were generally lower in comparison with current knowledge. In other words, at the lowest levels of desired knowledge (i.e., score of 1), attitudes were generally less positive than at the lowest levels of current knowledge or even slightly negative as occurred in the case of preregistrations and registered reports. We also observed that the relationship differed as a function of the considered perspective, *F* (2, 4,979) = 25.173, *p* < 0.001, ${\text{η}}_{p}^{2}$ = 0.009, with a significantly weaker association when attitudes referred to the public society, *β* = 0.26, 95% CI [0.22, 0.30], in comparison to ECRs’ daily research life, *β* = 0.37, 95% CI [0.33, 0.40], or their research field, *β* = 0.35, 95% CI [0.32, 0.39] (see Figure 6B).

#### 4.5.2. Knowledge and Perceived Need

**FIGURE 7:**
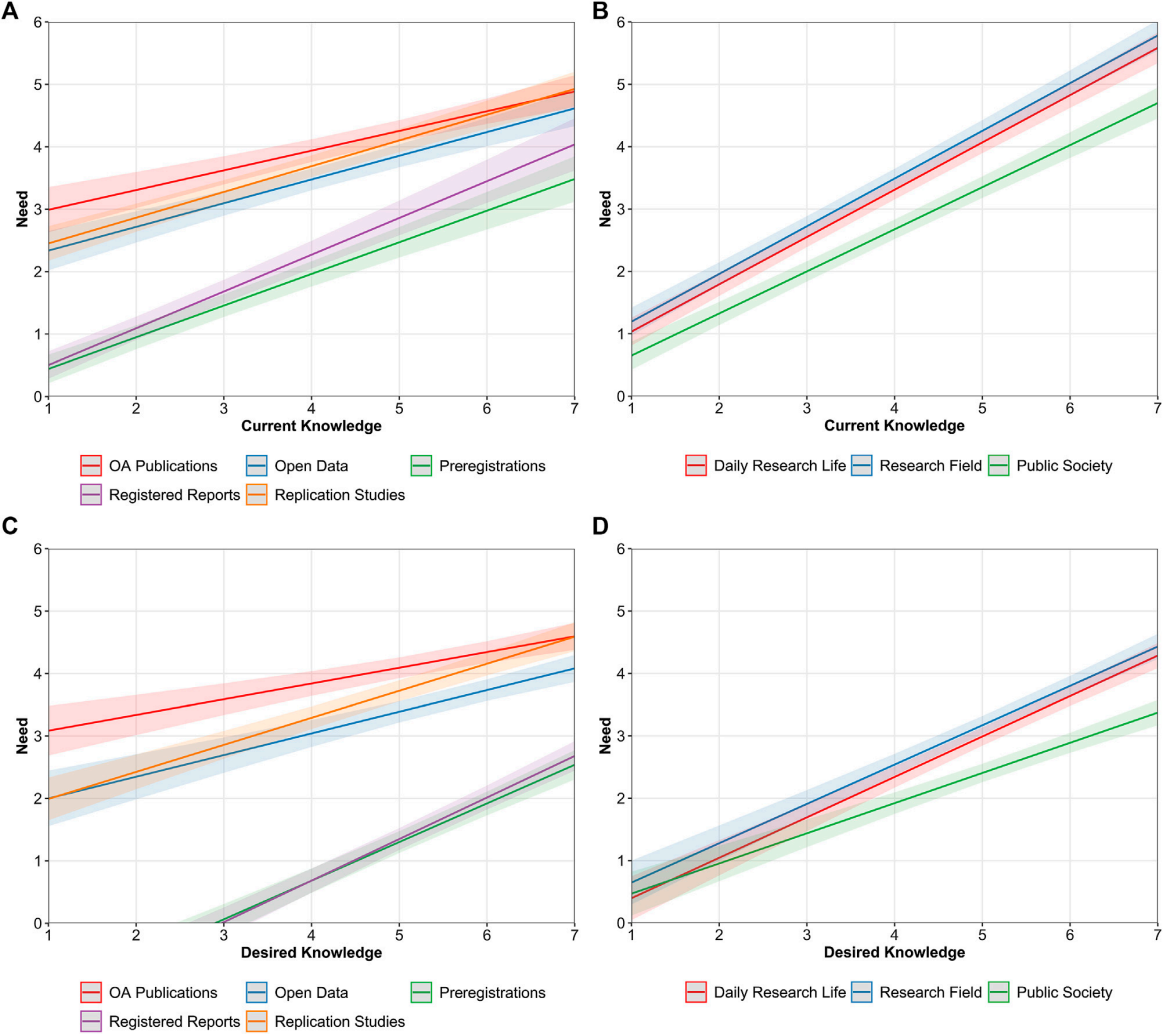
Predicted values of perceived need as a function of ECRs’ current knowledge across OS practices **(A)** and different perspectives **(B)** and as a function of desired knowledge across OS perspectives **(C)** and different perspectives **(D)**. Bandwidths represent 95% confidence intervals (CIs).

Closely resembling our findings about knowledge and attitudes, we observed similarly positive relationships between both types of knowledge (current and desired) and perceived need. The more ECRs knew or desired to know about a specific OS practice, the more they perceived it as necessary for their daily research life, their research field, and the public society. These relationships significantly differed across OS practices and across the different perspectives as were observed with attitudes. For a matter of conciseness, these results are not described in detail but presented in Figure 7 for further reference.

#### 4.5.3. Attitudes and Perceived Need

As could be inferred from the similar results regarding their association with knowledge, the attitudes that ECRs held toward OS practices were significantly positively associated with how necessary they perceived them, *F* (1, 6,560) = 17,159, *p* < 0.001, ${\text{η}}_{p}^{2}$ = 0.747. Although there were differences regarding this relationship across OS practices and the different perspectives for attitudes and need, the interaction effects were close to critical levels of statistical significance (*α* = 0.05) and were, therefore, not examined further.

#### 4.5.4. Implementation and Knowledge

The study of the relationship between the implementation of OS practices and ECRs’ knowledge was two staged. In two first models, we examined whether ECRs’ current and desired knowledge were associated to the past and future implementation of OS practices, respectively. Then, we focused on current knowledge, as it happened to be a better predictor of implementation of OS practices in both the past and the following 12 months and examined differences across OS practices.

The implementation of OS practices in the past 12 months was positively related to ECRs’ current knowledge, OR = 2.19, 95% CI [1.97, 2.44], but not with their desired knowledge, OR = 1.04, 95% CI [0.94, 1.14]. Current knowledge positively predicted the intention of future implementation of OS practices, OR = 2.13, 95% CI [1.95, 2.33], and was a stronger predictor compared to desired knowledge, OR = 1.37, 95% CI [1.27, 1.49]. Thus, our models estimated that with high levels of current knowledge (i.e., score of 7), 55% of ECRs would implement OS practices in the past and 82% in the future, while with high levels of desired knowledge, only predicted 8% would do so in the past and 39% in the future. These results suggest that the knowledge that ECRs had already acquired was a better predictor of the implementation of OS practices in the past and the future than their predisposition to acquire new knowledge.

**FIGURE 8:**
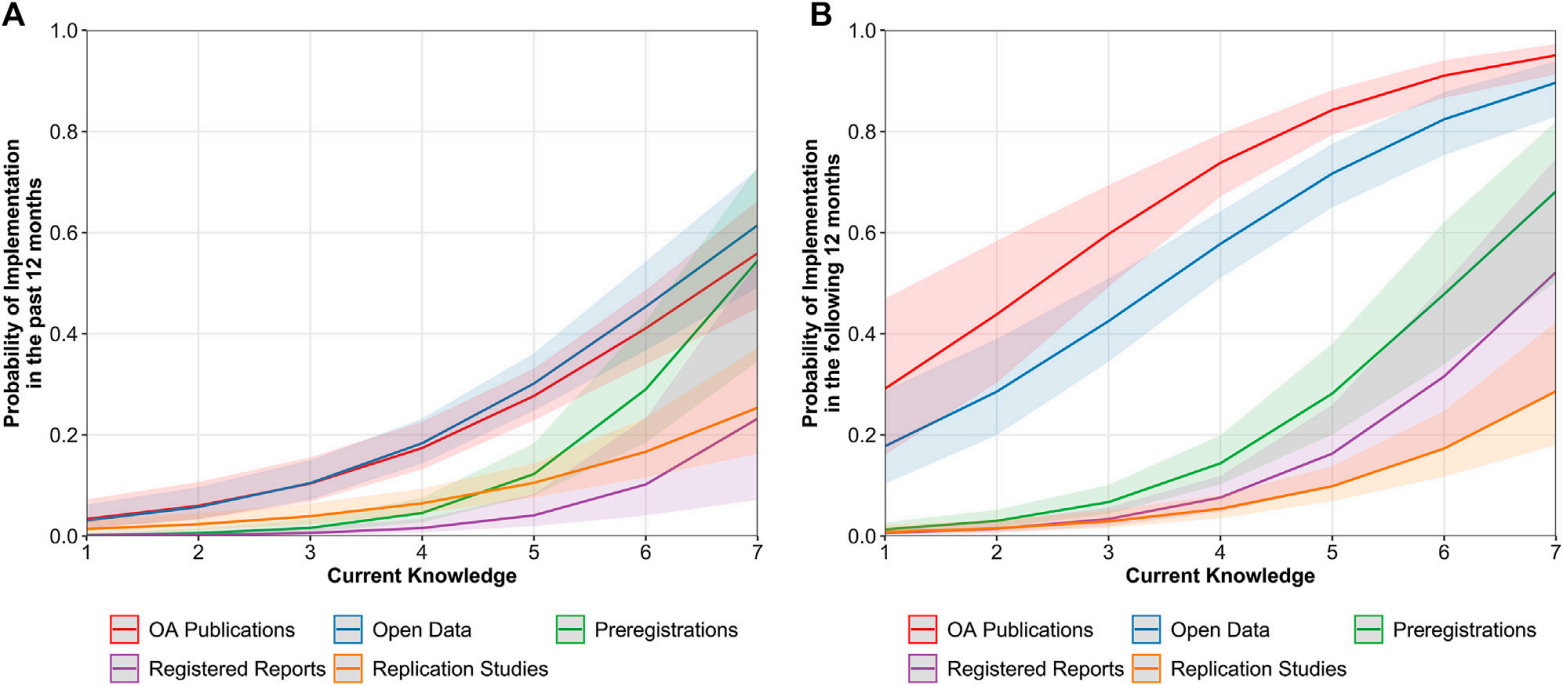
Predicted values of implementation in the past 12 months **(A)** and in the following 12 months **(B)** as a function of ECRs’ current knowledge across OS practices. Bandwidths represent 95% confidence intervals (CIs).

Since the role of ECRs’ current knowledge had more weight, we further examined its relationship with the past and future implementation across the different OS practices. As mentioned above, current knowledge positively predicted the implementation in the past 12 months of every OS practice but more pronouncedly in the case of preregistrations, OR = 2.93, 95% CI [2.14, 4.00], in comparison with OA publications, OR = 1.82, 95% CI [1.51, 2.19], open data, OR = 1.92, 95% CI [1.60, 2.31], and replication studies, OR = 1.70, 95% CI [1.39, 2.08], which did not significantly differ with each other. As for registered reports, we observed the broadest confidence interval, which entailed higher uncertainty about the actual strength of this positive relationship, OR = 2.66, 95% CI [1.66, 4.34]. However, it is noteworthy that even among those ECRs who reported to have a high current knowledge about the different OS practices, the level of implementation in the past 12 months would only reach 50–60% in the best case scenario (see Figure 8A). In contrast, the positive relationship between ECRs’ intentions to implement OS practices in the following 12 months and current knowledge did not significantly differ across OS practices. However, we observed that whereas these intentions were moderately present at low levels of current knowledge (i.e., score of 1) for OA publications (29%) and open data (18%), this was not the case for preregistrations, registered reports, and replication studies. For these three OS practices, intentions to implement them only appeared when ECRs had already acquired some knowledge about them (see Figure 8B).

#### 4.5.5. Implementation and Attitudes

For analyzing how attitudes and past and future implementation of OS practices were related, we also followed a twofold approach. We first examined whether ECRs’ average attitude across the different perspectives (i.e., mean of daily research life, research field, and public society) was associated to implementation and how this relationship differed across OS practices. Second, we tested whether attitudes reported while considering the different perspectives independently predicted the implementation of OS practices.

**FIGURE 9:**
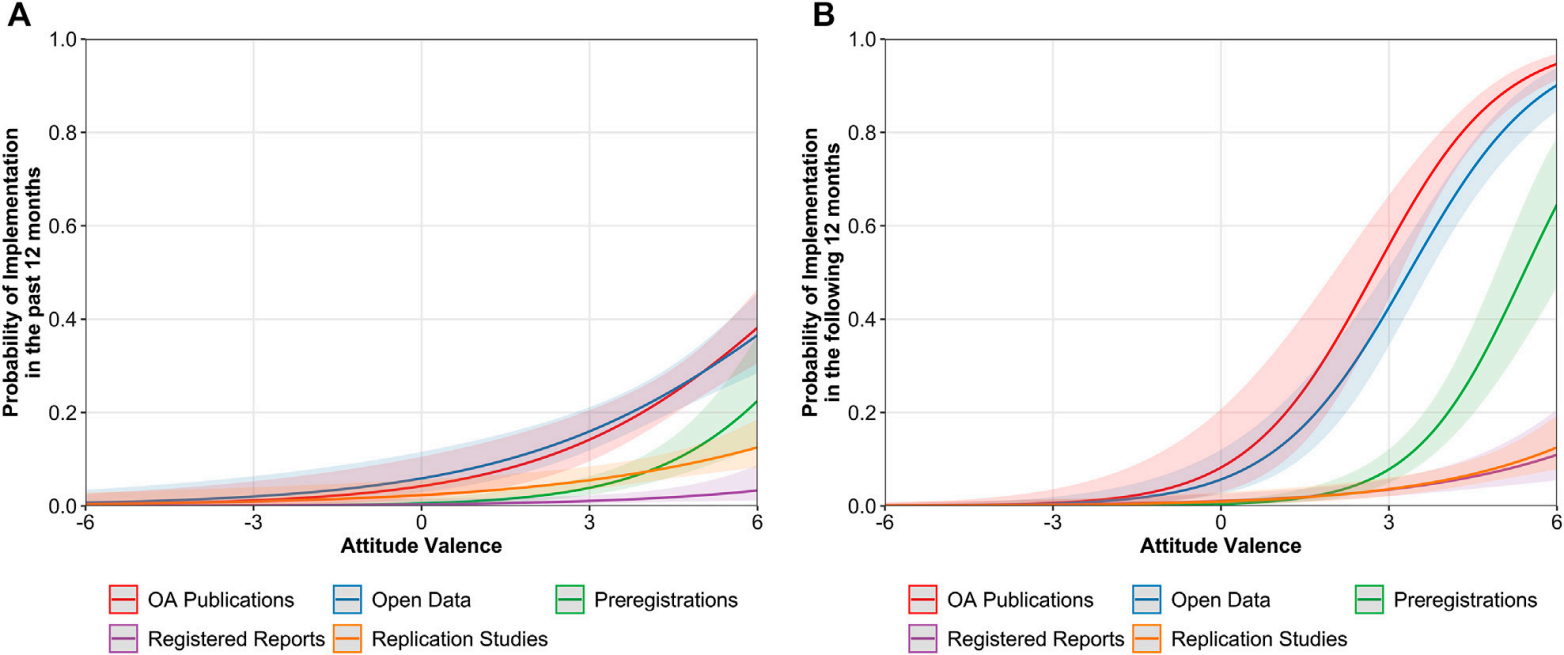
Predicted values of implementation in the past 12 months **(A)** and in the following 12 months **(B)** as a function of ECRs’ attitude valence across OS practices. Bandwidths represent 95% confidence intervals (CIs).

With regard to the implementation of OS practices in the past 12 months, it was positively predicted by the attitudes that ECRs held. However, this relationship differed across OS practices, being significantly more pronounced for preregistrations, OR = 1.93, 95% CI [1.50, 2.48], compared to open data, OR = 1.45, 95% CI [1.24, 1.70], and replication studies, OR = 1.35, 95% CI [1.12, 1.63]. OA publications, OR = 1.55, 95% CI [1.28, 1.88], and registered reports, OR = 1.47, 95% CI [1.03, 2.09], did not show statistically significant differences with any other OS practices (see Figure 9A). Furthermore, the implementation of OS practices in the past 12 months was independently predicted by attitudes considering the perspectives of ECRs’ daily research life, OR = 1.26, 95% CI [1.09, 1.46], their research field, OR = 1.31, 95% CI [1.12, 1.52], and less weakly by attitudes considering the perspective of the public society, OR = 1.08, 95% CI [1.01, 1.15].

Results regarding the intention to implement OS practices in the following 12 months showed that the positive relationship was significantly stronger for preregistrations, OR = 2.78, 95% CI [2.13, 3.61], OA publications, OR = 2.42, 95% CI [1.90, 3.07], and for open data, OR = 2.31, 95% CI [1.90, 2.81], than for registered reports, OR = 1.50, 95% CI [1.21, 1.87], and replication studies, OR = 1.57, 95% CI [1.25, 1.96]. Despite the strength of this relationship, we observe in Figure 9B that while extremely positive attitudes (i.e., score of 6) would respectively forecast 95 and 90% probability of implementation of OA publications and open data, 65% would be expected for preregistrations and only 5% for registered reports and replication studies. In terms of the considered perspectives, attitudes regarding the ECRs’ daily research life, OR = 1.42, 95% CI [1.26, 1.60], or research field, OR = 1.41, 95% CI [1.25, 1.60], significantly predicted the implementation of OS practices, while attitudes regarding the perspective of the public society did not, OR = 1.04, 95% CI [0.98, 1.10].

#### 4.5.6. Implementation and Perceived Need

Similarly to attitudes, we first examined whether ECRs’ average perceptions of need (i.e., across considered perspectives) were related to the implementation of the different OS practices in the past 12 months and the intentions to implement them in the following 12 months. Furthermore, we tested whether the perceptions of need reported while considering the different perspectives independently predicted implementation of OS practices in the past and the future.

**FIGURE 10:**
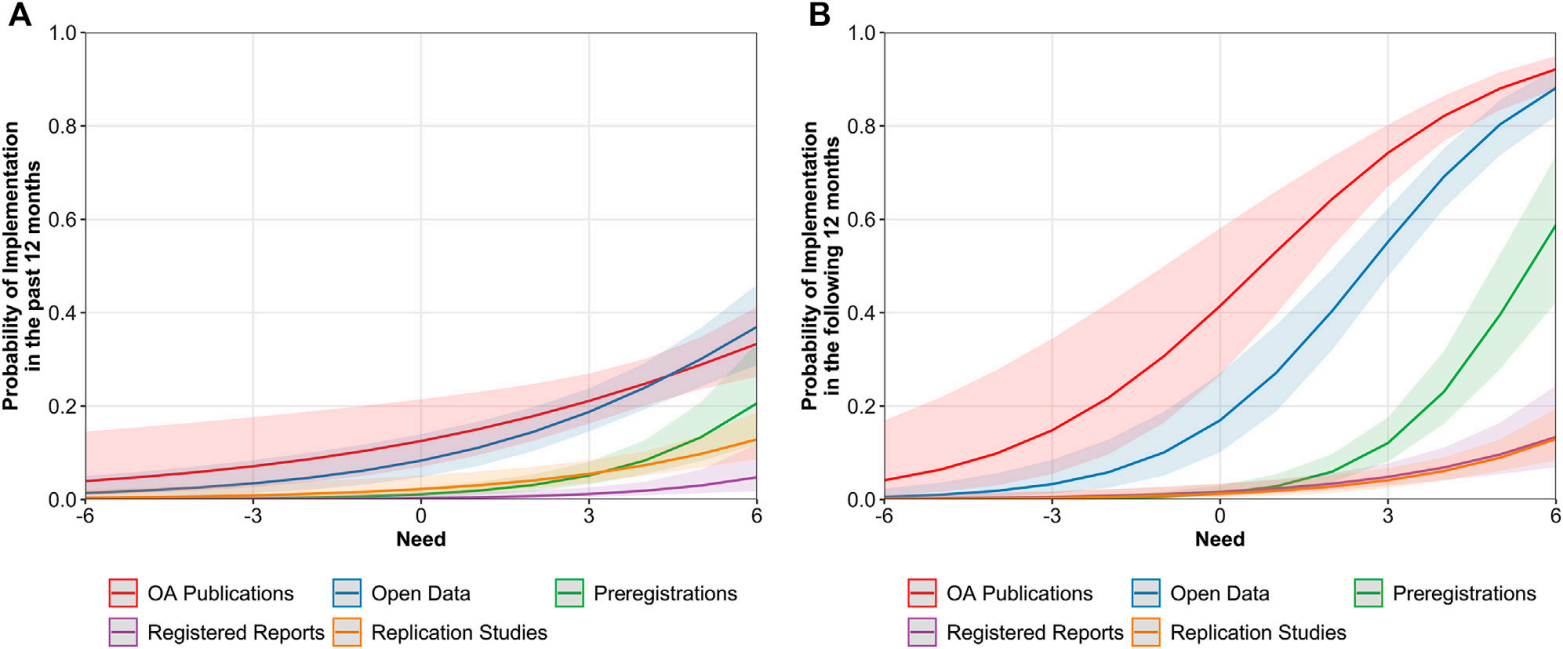
Predicted values of implementation in the past 12 months **(A)** and in the following 12 months **(B)** as a function of ECRs’ perceived need across OS practices. Bandwidths represent 95% confidence intervals (CIs).

The implementation of OS in the past 12 months was positively related to perceived need, but this positive relationship was significantly stronger for preregistrations, OR = 1.69, 95% CI [1.38, 2.07], than for OA publications, OR = 1.23, 95% CI [1.08, 1.41]. For open data, OR = 1.36, 95% CI [1.20, 1.55], registered reports, OR = 1.62, 95% CI [1.15, 2.28], and replication studies, OR = 1.37, 95% CI [1.15, 1.62], we did not observe significant differences in the strength of this relationship (see Figure 10A). Additionally, we observed that perceptions of need were independently predictive of past implementation when ECRs considered the perspective of their research field, OR = 1.25, 95% CI [1.11, 1.39], the perspective of their daily research life, OR = 1.12, 95% CI [1.01, 1.25], or the perspective of the public society, OR = 1.08, 95% CI [1.02, 1.15].

Focusing on the intention of ECRs to implement OS practices in the following 12 months, we observed that perceived need was significantly more predictive in the case of preregistrations, OR = 2.18, 95% CI [1.77, 2.68], than in the cases of OA publications, OR = 1.60, 95% CI [1.37, 1.87], registered reports, OR = 1.45, 95% CI [1.21, 1.74], and replication studies, OR = 1.51 and 95% CI [1.24, 1.85]. For open data, the relationship did not significantly differ from the other OS practices, OR = 1.82, 95% CI [1.57, 2.11]. Despite the strength of the relationship, Figure 10B shows that for OA publications and open data, high levels of perceived need led to 92 and 88% probability of implementing these OS practices in the future. For preregistration, this probability decreases to 59%, and for registered reports and replication studies, it drops to less than 20%. In addition, the intention to implement the different OS practices in the future was significantly predicted by the perceived need for ECRs’ daily research life, OR = 1.25, 95% CI [1.14, 1.37], and their research field, OR = 1.30, 95% CI [1.18, 1.42] but not by the need for the public society, OR = 1.02, 95% CI [0.97, 1.07].

## 5. Discussion

The digital age facilitates the connection of scientists worldwide and is an essential prerequisite for the OS movement. Since ECRs are essential future stakeholders of the OS movement, it is crucial to assess the status quo of their knowledge, attitude, and implementation regarding OS practices. In order to aid the development of new training strategies and creating incentives for facilitating a fast transition to an open scientific culture, we conducted a survey among the cross-disciplinary and international cohort of ECRs at the Max Planck Society. The results from 568 doctoral researchers revealed that ECRs have an overall positive predisposition toward the different OS practices analyzed in this survey, namely, OA publications, open data, preregistrations, registered reports, and replication studies. ECRs reported to be generally knowledgeable about these different practices, while systematically expressing a high interest in learning more about them. Moreover, ECRs held fairly positive attitudes toward the different OS practices and considered them to be generally necessary. The implementation of these practices was, however, not high in the 12 months before the survey was conducted, which is in stark contrast with the positive view that ECRs seemed to have of these practices. One could argue that this is partly due to the early stage of their academic career, in which most ECRs might not have had the opportunity to apply them yet (e.g., publish in an OA journal or openly share their research data). On the other hand, the levels of implementation found in this survey might be resembling actual levels of implementation in the German scientific community, as certainly occurred in the case of OA publications (i.e., 32% in 2019; see Open Access Monitor Project,^8^ Mittermaier et al., 2018). At the same time, we also observed that ECRs expressed high willingness to implement OS practices in the following 12 months. We consider that this intention, together with the generally positive attitudes that ECRs reported, portraits a rather fertile ground for the transition to a more open science. Nevertheless, some nuances must be considered with regard to the specific OS practices included in this survey, the different scientific sections to which ECRs belonged, and the distinct perspectives that ECRs were asked to consider when evaluating these practices (i.e., their daily research life, their research field, and the public society).

Across the OS practices examined in the present survey, we found consistent differences that might highlight the distinct prevalence of these OS practices in the scientific community. In general terms, preregistrations and registered reports were the OS practices which ECRs reported to have less knowledge and less positive attitudes about and which they also found less necessary. These two OS practices are tools against issues of reproducibility of scientific results (e.g., HARKing, p-hacking, and publication bias; Nosek et al., 2018; Yamada, 2018), which have had a greater incidence in specific research fields, such as the human and social sciences. This is reflected in our data, where preregistrations and registered reports were relatively more known, more positively evaluated, and more frequently used among ECRs from the HUM section than those from the BM and CPT sections. However, this could be likely to change in future years, with researchers from fields other than the social sciences calling for the implementation of preregistrations and registered reports to improve the transparency and reproducibility of their research fields (e.g., Parker et al., 2019; Walters et al., 2019). We further observed that ECRs across sections showed similarly low levels of participation in replication studies. One possible explanation for this can be rooted in the generalized, yet harmful, pressure for innovation and quantity over systematic reproduction and quality in scientific research. This is fostered by the so-called "publish or perish" culture within science, which particularly affects ECRs (Dalen and Henkens, 2012; Max Planck PhDnet Survey Group et al., 2019). Although the solution to this conundrum escapes the scope of the present research, we believe that one potential approach would be to fund more tangential and derivative research, which would guarantee that ECRs contribute to keep the foundations of the scientific enterprise solid and dependable. With regard to OA publications and open data, our results would closely resemble previous findings (Nicholas et al., 2017; Nicholas et al., 2019) by showing that generally ECRs hold a fairly positive stance toward these OS practices and that this, however, does not correspond with high levels of implementation. Despite that ECRs value OA publications and open data, these results suggest that external pressures and reputational concerns might limit the extent to which these practices are applied, such as the aim to publish in more prestigious journals with high-impact factors or the need to exploit a specific dataset before making it openly available to others (Nicholas et al., 2017; Nicholas et al., 2019).

While most ECRs agreed with the implementation of OS practices, we observed clear asymmetries between scientific sections. A consistent observation was that ECRs from the CPT section held a relatively less positive attitude about the different OS practices and were less interested in acquiring further knowledge about them, when compared with the BM and the HUM sections. A rather contradictory observation was that while ECRs from CPT held less positive attitudes toward OA publications, the highest prevalence of OA publications actually was within the CPT section. More concretely, ECRs from CPT showed higher self-archiving but lower formal OA publishing, when compared to the other two sections. Although we do not have a solid explanation for this, we speculate that the long self-archiving tradition in the field of physics might explain this higher prevalence, despite the difference in ECRs’ attitudes. At the same time, the less positive opinion and the lower interest to increase knowledge about OS practices of ECRs from CPT could speak of differences in the applicability of OS practices in specific research fields. The type of research conducted or data produced might hinder the application of specific OS practices, while still allowing for the publication of the results in an OA format. Moreover, different incentive systems between scientific communities and research fields might also explain the asymmetries in how ECRs think of and implement the discussed OS practices. For instance, one possible determinant of OA publications can be the OA agreements between academic institutions and field-specific publishers, which allow researchers to make their research openly accessible without compromising their research budget. Another example refers to OS practices intended to counter reproducibility issues. While in the human and social sciences, preregistrations and registered reports can be successful measures to deal with this problem (Nosek and Lakens, 2014; Nosek et al., 2018), other research fields, like artificial intelligence, might rely on promoting the open access of data as well as an incentive system that favors replication (Hutson, 2018).

It might not be surprising that ECRs generally showed a more positive opinion toward the different OS practices when considering the perspective of their daily research life and their research field than when thinking about the public society. As a matter of fact, this might reflect the specific utility of some of these OS practices for the improvement of science and individual research workflows, from which the general public might not directly benefit to the same extent. However, these results put into question whether one of the main goals of OS, namely, the free accessibility of science to the public, entails a priority for ECRs, at least, relatively to their direct scientific environment. We think this observation is especially relevant, and we will consider it below when discussing the practical implications of the present findings.

Our data also presented some gender differences. Female ECRs expressed higher desired knowledge about OS practices than men, which could have at least two possible interpretations. On the one hand, women might be more eager to acquire more knowledge about OS practices. On the other hand, men might feel on average more confident about their current knowledge on OS practices. Moreover, we found that women held a generally more positive opinion regarding preregistrations and registered reports compared to men. We suspect that the higher representation of women in the HUM section, where these practices have become more common, could be a plausible explanation of this last gender difference. Taken together, these gender differences might suggest a higher conscientiousness from female ECRs about the importance of the application of some of these OS practices in specific research fields. Nevertheless, due to the exploratory nature of the present survey, we believe that these gender differences should be interpreted cautiously. Before considering any potential implication, we consider it indispensable to reproduce similar results in further and more specific surveys.

The examination of the relationship between ECRs’ knowledge, attitudes, perceived need, and implementation of the OS practices also yielded useful insight. ECRs with higher current and desired knowledge tended to hold more positive opinions about OS practices and perceive them as more necessary. These three variables did, in turn, positively predict the likelihood of past and future implementation of each respective OS practice. Among them, the clearest predictor of whether ECRs had applied or intended to apply a specific OS practice was ECRs’ current knowledge about that practice, especially in the case of the less implemented OS practices (i.e., preregistrations, registered reports, and replication studies). Extending the conclusions drawn from previous work (Nicholas et al., 2017; Nicholas et al., 2019), the present findings indicate that those ECRs holding a positive predisposition toward OS practices (including OA publications and open data) are more likely to implement these practices. Importantly, even in the best case scenario in which ECRs were highly knowledgeable about an OS practice, held an extremely positive attitude toward it and perceived it as extremely necessary, the likelihood of implementation did not reach 100%. This underlines the importance of external factors which, beyond the individual predisposition of ECRs, facilitate the implementation of OS practices (e.g., supporting work environment, congruent incentive system, and a scientific culture aligned with OS). Furthermore, we observed that the association between ECRs’ opinions (i.e., attitudes and perceived need) with the implementation of OS practices (i.e., in the past and the future 12 months) was weaker or even null from the perspective of the public society. This questions again whether the potential repercussions of OS on the public society play a comparable role on the decision of ECRs to apply OS practices, relative to their most direct implications on daily research life and scientific field.

### 5.1. Limitations

The present work is not exempt from some limitations, mainly related to the representativeness of the analyzed sample.

First, the term ECR usually refers to both pre- and postdoctoral researchers with less than 10–12 years of experience from the beginning of their PhD program (with some differences across institutions, e.g., European Commission vs. Australian Research Council). Here, we exclusively focused on doctoral candidates due to matters of sample accessibility. We acknowledge that this could limit the implications of our findings to some extent. One would expect that postdoctoral ECRs have more nuanced opinions about OS practices in their respective fields and that they have had more opportunities to implement these practices due to their longer professional experience. Thus, the inclusion of data from postdoctoral ECRs would plausibly show even more pronounced differences between scientific sections and potentially a higher level of implementation. However, even despite the unique consideration of predoctoral ECRs, our data already show considerable differences between research fields and a large margin of improvement with regard to the implementation of the analyzed OS practices that is unlikely to completely vanish among postdoctoral ECRs.

A second consideration is that the exclusive focus on ECRs from the same research institution could compromise the generalization of our results in two respects. First, our sample mainly represented Germany-based ECRs and cannot capture the potential variation in views toward OS across different countries observed in previous work (Nicholas et al., 2017; Jamali et al., 2020). However, our findings are based on a substantially bigger sample than most previous studies (e.g., Stürmer et al., 2017; Nicholas et al., 2019) and, importantly, from a population of doctoral researchers in which roughly half are actually expatriates from multiple geographical backgrounds (see Max Planck PhDnet Survey Group et al., 2019; Regler et al., 2019). The second remarks regarding the exclusive focus on ECRs from the Max Planck Society are the international prestige of the latter and the role that this institution has with regard to OS (particularly, open access). With regard to the first, the Max Planck Society might offer more resources (i.e., funding, expert mentoring, etc.) than other academic institutions, which could partly explain some of our findings. For example, the current agreements that the Max Planck Digital Library holds with most publishers facilitate the publication of articles by Max Planck researchers in the OA format. This said, our data suggest that OS practices are not necessarily widespread, despite these favorable circumstances. As for the advocacy role of the Max Planck Society with regard to OS, we argued earlier that the decentralized organization of the Max Planck Society does not allow to assume that its organizational vision and actions (e.g., Max Planck Digital Library White Paper, Schimmer et al., 2015) affect the attitudes toward OS of every ECR. On the other hand, we cannot ascertain that ECRs in the Max Planck Society are differently exposed to pro-OS views in comparison to ECRs from other institutions.

A third factor that could affect the representativeness of our results was the self-selection bias that the voluntary participation in our survey could have introduced. That is, ECRs with positive attitudes and high interest toward OS could have been more responsive to our survey than those with less positive attitudes or interest. It is difficult to overcome this issue without data from a broader population of ECRs that does not suffer from the same issue (e.g., Jamali et al., 2020), which, to our knowledge, is not currently available in the literature. Possible solutions would include a meta-analytic review of available published and unpublished data or a multi-institutional collaborative project, which would allow for a greater sample size, more geographical representativeness, and a potential minimization of the self-selection bias.

Lastly, with regard to the scope of our analyses, we previously acknowledged that the use of broad scientific sections as a unit of comparison might have not offered the most fine-grained picture of potential differences between specific research fields. In addition, most ECRs in the Max Planck Society conduct highly interdisciplinary research and, therefore, could have reported to belong to a scientific section that does not correspond to their educational background but to the research conducted at their affiliating Max Planck Institute. Although one way to solve this issue would have been to assess ECRs’ specific research discipline or use their Max Planck Institute as unit of analysis, these solutions would have affected the statistical power of our analyses since the number of observation per discipline or research institute would have been insufficient in many cases. Taking this into account, the present work does not offer clear-cut comparisons between specific research fields but sheds light on important disciplinary differences and similarities between clusters of scientific disciplines closely related in the form of interdisciplinary research (e.g., biology and medicine or psychology and behavioral economics).

### 5.2. Implications for the Development of OS Policies and Training Programs

Regardless of the aforementioned limitations, we believe that the present work has clear implications for the transition to a more open scientific culture, which will only be successful if the proper OS policies are put into place by governments, academic institutions, and funding agencies. Our survey highlights the crucial necessity of promoting further training on the benefits and risks of OS practices, as others have previously suggested (Schönbrodt, 2019). According to our findings, this would not only increase the knowledge of ECRs about specific OS practices but also could foster its implementation among ECRs. We believe that it would already be highly beneficial to introduce these training schemes in the curriculum of undergraduates programs, as well as in courses on good scientific practices that many ECRs have to complete when joining their first affiliating institution. These courses should clearly cover the benefits and risks of OS practices, together with a guideline on how to implement them (for an example, see Farnham et al., 2017). However, it is also important to take one step back and acknowledge that OS still requires the establishment of clear guidelines for transparency and openness of research at the international level. Examples for guidelines for OA publishing (Nosek et al., 2015; Schiltz, 2018), FAIR data management (Wilkinson et al., 2016), as well as OS collaborations (Gold et al., 2019) are already existing, and their use should be promoted by governments and funding agencies, as well as integrated in the training of ECRs by academic institutions. In this line, Nicholas et al. (2019, 2020) point out that organizations and/or regulators in charge of overviewing the open scholarly system need to be established. We further argue that these organizations need to be coordinated to avoid the application of inconsistent OS policies around the globe.^9^ This is not an easy endeavor, given the barriers that these policies often find in important legal frameworks that drastically differ across geographical areas (e.g., those related to data protection or intellectual property; Ali-Khan et al., 2018). However, efforts in this direction start being visible. One example is the recently proposed “European Open Science Cloud” (EOSC) initiative, which intends to create the infrastructure and standards defining a European FAIR data-sharing framework, while considering its synergies with initiatives from other international partners (European Commission, 2020). In short, we emphasize that the importance of the aforementioned issues cannot be underestimated and that it might require international efforts to create common OS standards that place ECRs’ training high on the agenda.

An additional factor to foster the implementation of OS practices is that every stakeholder, including governments, academic institutions, and funding agencies, provides aligned incentive systems to promote OS. However, how to provide these incentives seems to be the rate-limiting step for the OS movement. For example, while funding agencies already require publication of findings in OA schemes and data-sharing plans (Neylon, 2017), it could be argued that the weight that OS currently has for researchers’ career advancement is rather small. Hence, in addition to compulsory requirements from funders, which might only lead researchers to show minimal compliance (Neylon, 2017), individual incentives for researchers should be introduced through, for example, professional recognition or the allocation of extra funding (Kidwell et al., 2016; Fecher et al., 2015; Ali-Khan et al., 2018). Our data indirectly suggest that in those fields where a specific OS practice is more established and recognized, ECRs implement it more (e.g., preregistrations in the HUM section). Previous work indicates that ECRs may be afraid of jeopardizing their career when implementing OS practices (Nicholas et al., 2017). At the same time, they might feel that investing additional effort in making research open (i.e., transparent and reproducible) is unrewarded (Nicholas et al., 2017), such as conducting replication studies that might not be considered for publication in high-impact journals. Thus, it seems fundamental to counter these concerns by stressing the benefits that OS can have for ECRs. Based on our data, we consider especially important to emphasize how OS practices can improve ECRs’ daily research life and research field to promote their implementation. For example, OA publications have been found to receive more citations than paywalled publications (Piwowar et al., 2018) and can therefore aid ECRs’ career advancement. Similarly, open data can be highly beneficial to promote new collaborations and increase the number of citations and the confidence that the field has in the findings (Popkin, 2019). Another promising incentive to increase the use of OS practices might be to establish OS as a hiring criterion, such as promoted in the San Francisco Declaration on Research Assessment.^10^ The first hiring institutions that require an OS statement from applicants to leading positions in academia are already pioneering this transition (Schönbrodt, 2019). Last but not least, we consider it critical to highlight the impact that OS practices might have for the public society. This includes the open access of publicly funded research (OECD, 2015) but additionally, the necessary reinforcement of public trust in science that OS may help to promote (Rutjens et al., 2018). In this regard, further empirical contributions to the current literature are necessary to measure and comprehend the societal effects of OS beyond academia and could potentially be used to strengthen the attitudes of the scientific community toward OS.

## 6. Conclusion and Final Remarks

In conclusion, the present survey offers an optimistic picture of the readiness of ECRs to facilitate the transition to OS. However, it also reveals the need to introduce smart OS policies with clear incentives and specific training strategies that guide this impulse from young researchers. This involves the different stakeholders that confirm the already established academic system, among them, governments, scientific institutions, and funding agencies. The heterogeneity between scientific fields should be considered when applying these policies with the aim of fostering specific OS practices. In this respect, future surveys should clarify which specific incentives and obstacles might affect the decision of ECRs to implement a concrete OS practice in their research and field. In addition, longitudinal surveys would not only help to provide insightful information about the stance of ECRs toward OS but also could further assess the concrete effects of newly implemented OS policies and training programs on OS.

## Data Availability Statement

The dataset presented in this study, together with its code for data analysis and research material, can be found in the following online repository: https://osf.io/qbgct/

## Author Contributions

All authors were involved in the development of the conceptual framework of the investigation. All authors contributed to the study design and IT implemented the online survey. DT-F, LA, and MP performed the data analyses, data visualization, and the interpretation of the results. DT-F, LA, MP, IT, and FND contributed to drafting the manuscript, while HW provided critical revisions. All authors agreed to the final version of the submitted manuscript.

## Conflict of Interest

The authors declare that the research was conducted in the absence of any commercial or financial relationships that could be construed as a potential conflict of interest.

## Footnotes

1
https://www.budapestopenaccessinitiative.org/

2
https://openaccess.mpg.de/67605/berlin_declaration_engl.pdf

3
https://lyondeclaration.org/

4
https://www.coalition-s.org/

5
https://oa2020.org/

6
https://www.cos.io/

7
https://www.budapestopenaccessinitiative.org/

8
https://open-access-monitor.de/

9 Examples of OS policies across different continents: the EU Open Science Policy Platform (OSPP), the Australian Research Data Commons (ARDC), the African Open Science Platform (AOSP), the US National Institutes of Health (NIH) Data Commons, Canada's National Data Services Framework (NDSF), the China Science and Technology (CST) Cloud, or the Association of Southeast Asian Nations (ASEAN) initiative (European Commission, 2020).

10
https://sfdora.org/
