# Supplementary material for: Where Do Early Career Researchers Stand on Open Science Practices? A Survey Within the Max Planck Society
Source: Front Res Metr Anal. 2021 Jan 22;5:586992. doi: 10.3389/frma.2020.586992 (PMC8025980; doi:10.3389/frma.2020.586992)
Supplement: Supplementary file 1 [file datasheet1.pdf]

Supplementary Material

This document contains the parts of the Survey presented in the research article.

Informed Consent

“This is a voluntary survey for all researchers working in the Max Planck Society. The survey aims at providing PhDnet and all researchers with a clear picture of the knowledge, attitude, need and implementation of Open Science and Good Scientific Practises of researchers of the Max Planck Society.

Please take time and carefully read the following terms and conditions regarding data protection and usage: The data you provide in this survey will only be evaluated and presented in an aggregated and anonymized form. All the data collected comply with the General Data Protection Regulation (GDPR).It is not possible to identify you at any moment, as long as you refrain from providing personal data. The aggregated and anonymized data will be shared with the PhDnet Steering Group, the Max Planck Digital Library and the Max Planck General Administration. The External PhD Representatives will be informed, when the final survey report is online, where it can also be downloaded by all researchers.

In addition, the anonymized data will potentially become openly available through its publication in scientific journals that endorse Open Access standards. The survey is hosted on LimeSurvey with restricted access provided by the Max Planck Society. Participating in the survey is possible via a unique token generating system. If you stop participating in the survey before completion, the collected data will be automatically deleted. In order for the survey to provide representative data, it is essential that the majority of researchers of the Max Planck Society participate.

The questionnaire will take about 10 to 15 minutes to complete and contains questions about scientific practices, open publishing, open data and confirmatory research. The final report will be made available at the end of 2019 at https://www.phdnet.mpg.de/home. If you have questions, or wish to report technical issues, you can reach any of the following members of the PhDnet OpenScience Working Group:

Isabell Tunn

Daniel Toribio-Florez

If you click "Next", you accept the terms and conditions listed above, as well as the use of the provided data in case you complete the survey.”

Section 1 - Open Science Practices

In this section, we assessed the opinion about different scientific practices related to Open Science. We first presented a brief description of a specific scientific practice. The definitions used for each Open Science practice are presented below:

Open Access Publications: Unrestricted public availability of a research paper on the internet through formal publication systems (e.g. Open Access Publishers) or author self-archiving (e.g. working papers or pre-prints in online repositories).

Open Data: Unrestricted public availability of research data and/or any resource necessary for the collection of this data (methodology, protocol, software packages, etc.), generally through online repositories.

Pre-registrations: Detailed registration of hypotheses, study design, methodology and analyses prior to data collection to which the researcher commits in order to guarantee a transparent confirmatory (vs. exploratory) test of the formulated hypotheses.

Registered Reports: Publication format consisting of two peer-reviewed stages: 1) An in-principle acceptance of a research paper prior to data collection based on a detailed preregistration, and 2) The actual publication of the research paper after data collection, including any result (statistically significant or not) obtained by following what was specified in the preregistration.

Replication Studies: Studies intended to 1) reproduce a scientific finding from a previous study by recreating the critical elements that are assumed to explain the original result (i.e., close replication), or 2) generalize a scientific finding by purposefully modifying at least one component of the original study, such as the sample, the protocol, or the material used (i.e., conceptual replication).

Most questions following the definitions used a 7-point scale (1 – Not at all, 7 – Extremely), and included the option “I don’t want to answer this question.

Open Access Publications:

Definition: Unrestricted public availability of a research paper on the internet through formal publication systems (e.g. Open Access Publishers) or author self-archiving (e.g. working papers or pre-prints in online repositories).

1: "To what extent are you knowledgeable about Open Access Publications?"

Type: single choice

1-not at all (n=14), 2 (n=29), 3 (n=74), 4 (n=109), 5 (n=159), 6 (n=132), 7-extremely (n=51), I don't want to answer this question (n=0)

Q2: "To what extent would you like to know more about Open Access Publications?"

Type: single choice

1-not at all (n=11), 2 (n=23), 3 (n=48), 4 (n=63), 5 (n=118), 6 (n=170), 7-extremely (n=135), I don't want to answer this question (n=0)

Q3: "For your daily life as a researcher, to what extent do you think that Open Access Publications are/can be...

Type for each subquestion: single choice

…useful?"

1-not at all (n=0), 2 (n=5), 3 (n=7), 4 (n=21), 5 (n=57), 6 (n=122), 7-extremely (n=352), I don't want to answer this question (n=4)

…useless?"

1-not at all (n=435), 2 (n=77), 3 (n=25), 4 (n=13), 5 (n=5), 6 (n=3), 7-extremely (n=4), I don't want to answer this question (n=6)

…advantageous?"

1-not at all (n=1), 2 (n=3), 3 (n=7), 4 (n=29), 5 (n=65), 6 (n=160), 7-extremely (n=298), I don’t want to answer this question (n=5)

…disadvantageous?”

1-not at all (n=385), 2 (n=102), 3 (n=37), 4 (n=12), 5 (n=8), 6 (n=3), 7-extremely (n=1), I don’t want to answer this question (n=10)

…beneficial?”

1-not at all (n=1), 2 (n=2), 3 (n=10), 4 (n=27), 5 (n=69), 6 (n=149), 7-extremely (n=304), I don’t want to answer this question (n=6)

…harmful?”

1-not at all (n=362), 2 (n=107), 3 (n=44), 4 (n=30), 5 (n=12), 6 (n=4), 7-extremely (n=0), I don’t want to answer this question (n=9)

…necessary?”

1-not at all (n=3), 2 (n=9), 3 (n=31), 4 (n=47), 5 (n=88), 6 (n=124), 7-extremely (n=260), I don’t want to answer this question (n=6)

…unnecessary?”

1-not at all (n=382), 2 (n=95), 3 (n=39), 4 (n=26), 5 (n=16), 6 (n=4), 7-extremely (n=2), I don’t want to answer this question (n=10)

Q4: “For your research field, to what extent do you think that Open Access Publications are/can be...

Type for each subquestion: single choice

…useful?”

1-not at all (n=0), 2 (n=6), 3 (n=5), 4 (n=23), 5 (n=62), 6 (n=133), 7-extremely (n=332), I don't want to answer this question (n=7)

…useless?"

1-not at all (n=426), 2 (n=78), 3 (n=29), 4 (n=18), 5 (n=8), 6 (n=1), 7-extremely (n=1), I don't want to answer this question (n=10)

…advantageous?"

1-not at all (n=0), 2 (n=3), 3 (n=10), 4 (n=28), 5 (n=65), 6 (n=146), 7-extremely (n=307), I don't want to answer this question (n=9)

…disadvantageous?"

1-not at all (n=394), 2 (n=84), 3 (n=45), 4 (n=18), 5 (n=9), 6 (n=3), 7-extremely (n=0), I don't want to answer this question (n=15)

…beneficial?"

1-not at all (n=1), 2 (n=1), 3 (n=14), 4 (n=22), 5 (n=71), 6 (n=148), 7-extremely (n=304), I don't want to answer this question (n=7)

…harmful?"

1-not at all (n=380), 2 (n=85), 3 (n=48), 4 (n=30), 5 (n=10), 6 (n=3), 7-extremely (n=0), I don't want to answer this question (n=12)

…necessary?"

1-not at all (n=3), 2 (n=12), 3 (n=17), 4 (n=53), 5 (n=86), 6 (n=114), 7-extremely (n=274), I don't want to answer this question (n=9)

…unnecessary?"

1-not at all (n=380), 2 (n=75), 3 (n=47), 4 (n=39), 5 (n=8), 6 (n=5), 7-extremely (n=0), I don't want to answer this question (n=14)

Q5: “For the public society, to what extent do you think that Open Access Publications are/can be...

Type for each subquestion: single choice

…useful?”

1-not at all (n=1), 2 (n=13), 3 (n=20), 4 (n=36), 5 (n=90), 6 (n=107), 7-extremely (n=294), I don’t want to answer this question (n=7)

…useless?”

1-not at all (n=354), 2 (n=102), 3 (n=42), 4 (n=30), 5 (n=16), 6 (n=8), 7-extremely (n=2), I don’t want to answer this question (n=14)

…advantageous?”

1-not at all (n=3), 2 (n=11), 3 (n=21), 4 (n=43), 5 (n=79), 6 (n=128), 7-extremely (n=274), I don’t want to answer this question (n=9)

…disadvantageous?”

1-not at all (n=375), 2 (n=102), 3 (n=36), 4 (n=27), 5 (n=6), 6 (n=5), 7-extremely (n=1), I don’t want to answer this question (n=16)

…beneficial?”

1-not at all (n=2), 2 (n=11), 3 (n=22), 4 (n=48), 5 (n=72), 6 (n=123), 7-extremely (n=282), I don’t want to answer this question (n=8)

…harmful?”

1-not at all (n=363), 2 (n=95), 3 (n=36), 4 (n=32), 5 (n=16), 6 (n=8), 7-extremely (n=5), I don’t want to answer this question (n=13)

…necessary?”

1-not at all (n=9), 2 (n=21), 3 (n=35), 4 (n=70), 5 (n=81), 6 (n=103), 7-extremely (n=242), I don’t want to answer this question (n=7)

…unnecessary?”

1-not at all (n=341), 2 (n=100), 3 (n=46), 4 (n=43), 5 (n=11), 6 (n=10), 7-extremely (n=2), I don’t want to answer this question (n=15)

Q6: “In the last 12 months, have you published Open Access any of your research papers?”

Type: single choice

Answer options:

Yes (n=170)

No (n=373)

I don’t want to answer this question (n=25)

Q6.1: Conditional question if Q6 = Yes: “Where have you published it?”

Type: single choice (n= 170)

Answer options:

Open Access Publisher (n=109)

Self-archiving (n=30)

Other (n=25)

No answer (n=6)

Q7: “In the following 12 months, are you planning to publish Open Access any of your research papers?”

Type: single choice

Answer options:

Yes (343)

No (125)

I don’t want to answer this question (100)

Q7.1: Conditional question if Q7 = Yes: “Where are you planning to publish it?”

Type: single choice (n=343)

Answer options:

Open Access Publisher (210)

Self-archiving (n=62)

Other (n=32)

No answer (n=39)

Q8: ”If you feel that the previous questions did not completely capture your opinion about Open Access Publications, you can use the following blank for developing your thoughts:”

Type: free text

The comments are not reported for reasons of brevity and anonymity of the respondents.

Open Data

Definition: Unrestricted public availability of research data and/or any resource necessary for the collection of this data (methodology, protocol, software packages, etc.), generally through online repositories.

Q9: “To what extent are you knowledgeable about Open Data?”

Type: single choice

Answer options:

1-not at all (=36), 2 (n=55), 3 (n=87), 4 (n=128), 5 (n=153), 6 (n=77), 7-extremely (n=31), I don’t want to answer this question (n=1)

Q10: “To what extent would you like to know more about Open Data?”

Type: single choice

Answer options:

1-not at all (n= 8), 2 (n=16), 3 (n=30), 4 (n=72), 5 (n=119), 6 (n=170), 7-extremely (n=152), I don’t want to answer this question (n=1)

Q11: “For your daily life as a researcher, to what extent do you think that Open Data are/can be...

Type for each subquestion: single choice

…useful?”

1-not at all (n=3), 2 (n=6), 3 (n=11), 4 (n=40), 5 (n=74), 6 (n=127), 7-extremely (n=292), I don’t want to answer this question (n=15)

…useless?”

1-not at all (n=388), 2 (n=95), 3 (n=37), 4 (n=18), 5 (n=5), 6 (n=3), 7-extremely (n=1), I don't want to answer this question (n=21)

…advantageous?"

1-not at all (n=2), 2 (n=11), 3 (n=9), 4 (n=35), 5 (n=89), 6 (n=134), 7-extremely (n=272), I don't want to answer this question (n=16)

…disadvantageous?"

1-not at all (n=343), 2 (n=122), 3 (n=52), 4 (n=23), 5 (n=9), 6 (n=7), 7-extremely (n=3), I don't want to answer this question (n=20)

…beneficial?"

1-not at all (n=4), 2 (n=4), 3 (n=12), 4 (n=44), 5 (n=84), 6 (n=142), 7-extremely (n=262), I don't want to answer this question (n=16)

…harmful?"

1-not at all (n=321), 2 (n=100), 3 (n=63), 4 (n=40), 5 (n=14), 6 (n=7), 7-extremely (n=3), I don't want to answer this question (n=20)

…necessary?"

1-not at all (n=4), 2 (n=16), 3 (n=26), 4 (n=67), 5 (n=98), 6 (n=115), 7-extremely (n=221), I don't want to answer this question (n=21)

…unnecessary?"

1-not at all (n=347), 2 (n=100), 3 (n=50), 4 (n=35), 5 (n=7), 6 (n=2), 7-extremely (n=1), I don't want to answer this question (n=25)

Q12: "For your research field, to what extent do you think that Open Data are/can be...

Type for each subquestion: single choice

…useful?"

1-not at all (n=1), 2 (n=8), 3 (n=8), 4 (n=34), 5 (n=66), 6 (n=113), 7-extremely (n=321), I don't want to answer this question (n=17)

…useless?"

1-not at all (n=392), 2 (n=100), 3 (n=29), 4 (n=13), 5 (n=4), 6 (n=3), 7-extremely (n=2), I don't want to answer this question (n=25)

…advantageous?"

1-not at all (n=2), 2 (n=6), 3 (n=12), 4 (n=32), 5 (n=68), 6 (n=128), 7-extremely (n=299), I don't want to answer this question (n=21)

…disadvantageous?"

1-not at all (n=360), 2 (n=105), 3 (n=43), 4 (n=17), 5 (n=6), 6 (n=9), 7-extremely (n=2), I don't want to answer this question (n=26)

…beneficial?"

1-not at all (n=2), 2 (n=6), 3 (n=10), 4 (n=38), 5 (n=71), 6 (n=127), 7-extremely (n=294), I don't want to answer this question (n=20)

…harmful?"

1-not at all (n=331), 2 (n=101), 3 (n=59), 4 (n=27), 5 (n=9), 6 (n=9), 7-extremely (n=3), I don't want to answer this question (n=26)

…necessary?"

1-not at all (n=5), 2 (n=14), 3 (n=21), 4 (n=53), 5 (n=78), 6 (n=117), 7-extremely (n=255), I don't want to answer this question (n=24)

…unnecessary?”

1-not at all (n=357), 2 (n=99), 3 (n=42), 4 (n=31), 5 (n=7), 6 (n=2), 7-extremely (n=2), I don’t want to answer this question (n=28)

Q13: “For the public society, to what extent do you think that Open Data are/can be...

Type for each subquestion: single choice

…useful?”

1-not at all (n=14), 2 (n=43), 3 (n=47), 4 (n=80), 5 (n=124), 6 (n=87), 7-extremely (n=153), I don’t want to answer this question (n=20)

…useless?”

1-not at all (n=240), 2 (n=94), 3 (n=88), 4 (n=51), 5 (n=36), 6 (n=20), 7-extremely (n=10), I don’t want to answer this question (n=29)

…advantageous?”

1-not at all (n=13), 2 (n=35), 3 (n=44), 4 (n=94), 5 (n=104), 6 (n=103), 7-extremely (n=149), I don’t want to answer this question (n=26)

…disadvantageous?”

1-not at all (n=288), 2 (n=103), 3 (n=66), 4 (n=49), 5 (n=17), 6 (n=7), 7-extremely (n=6), I don’t want to answer this question (n=32)

…beneficial?”

1-not at all (n=14), 2 (n=28), 3 (n=52), 4 (n=81), 5 (n=112), 6 (n=101), 7-extremely (n=157), I don't want to answer this question (n=23)

…harmful?"

1-not at all (n=275), 2 (n=98), 3 (n=71), 4 (n=53), 5 (n=16), 6 (n=15), 7-extremely (n=9), I don't want to answer this question (n=31)

…necessary?"

1-not at all (n=29), 2 (n=44), 3 (n=60), 4 (n=100), 5 (n=89), 6 (n=87), 7-extremely (n=132), I don't want to answer this question (n=27)

…unnecessary?"

1-not at all (n=236), 2 (n=108), 3 (n=64), 4 (n=71), 5 (n=24), 6 (n=22), 7-extremely (n=11), I don't want to answer this question (n=32)

Q14: "In the last 12 months, have you made any of your research data and/or any of your methodological resources openly available?"

Type: single choice

Answer options:

Yes (n=153)

No (N=389)

I don't want to answer this question (n=25)

Q15:" In the following 12 months, are you planning to make any research data and/or any methodological resource openly available?"

Type: single choice

Answer options:

Yes (n=281)

No (n=211)

I don’t want to answer this question (n=76)

Q16: “If you feel that the previous questions did not completely capture your opinion about Open Data, you can use the following blank for developing your thoughts:”

Type: free text

The comments are not reported for reasons of brevity and anonymity of the respondents.

Pre-registrations

Definition: Detailed registration of hypotheses, study design, methodology and analyses prior to data collection to which the researcher commits in order to guarantee a transparent confirmatory (vs. exploratory) test of the formulated hypotheses.

Q17: "To what extent are you knowledgeable about Pre-registrations?"

Type: single choice

Answer options:

1-not at all (n=234), 2 (n=81), 3 (n=73), 4 (n=63), 5 (n=59), 6 (n=36), 7-extremely (n=10), I don't want to answer this question (n=12)

Q18: "To what extent would you like to know more about Pre-registrations?"

Type: single choice

Answer options:

1-not at all (n=19), 2 (n=37), 3 (n=48), 4 (n=83), 5 (n=114), 6 (n=114), 7-extremely (n=141), I don't want to answer this question (n=12)

Q19: "For your daily life as a researcher, to what extent do you think that Pre-registrations are/can be...

Type for each subquestion: single choice

…useful?"

1-not at all (n=24), 2 (n=39), 3 (n=43), 4 (n=94), 5 (n=109), 6 (n=70), 7-extremely (n=89), I don't want to answer this question (n=100)

…useless?"

1-not at all (n=180), 2 (n=70), 3 (n=81), 4 (n=60), 5 (n=35), 6 (n=26), 7-extremely (n=6), I don't want to answer this question (n=110)

…advantageous?"

1-not at all (n=21), 2 (n=38), 3 (n=39), 4 (n=109), 5 (n=101), 6 (n=77), 7-extremely (n=78), I don't want to answer this question (n=105)

…disadvantageous?"

1-not at all (n=160), 2 (n=73), 3 (n=77), 4 (n=86), 5 (n=32), 6 (n=18), 7-extremely (n=10), I don't want to answer this question (n=112)

…beneficial?"

1-not at all (n=19), 2 (n=38), 3 (n=39), 4 (n=97), 5 (n=111), 6 (n=78), 7-extremely (n=81), I don't want to answer this question (n=105)

…harmful?"

1-not at all (n=187), 2 (n=58), 3 (n=75), 4 (n=77), 5 (n=37), 6 (n=16), 7-extremely (n=5), I don't want to answer this question (n=113)

…necessary?"

1-not at all (n=40), 2 (n=68), 3 (n=46), 4 (n=104), 5 (n=76), 6 (n=59), 7-extremely (n=70), I don't want to answer this question (n=105)

…unnecessary?"

1-not at all (n=166), 2 (n=61), 3 (n=61), 4 (n=78), 5 (n=45), 6 (n=27), 7-extremely (n=16), I don't want to answer this question (n=114)

Q20: “For your research field, to what extent do you think that Pre-registrations are/can be...

Type for each subquestion: single choice

…useful?”

1-not at all (n=23), 2 (n=36), 3 (n=32), 4 (n=91), 5 (n=90), 6 (n=90), 7-extremely (n=98), I don’t want to answer this question (n=108)

…useless?”

1-not at all (n=188), 2 (n=74), 3 (n=59), 4 (n=75), 5 (n=29), 6 (n=19), 7-extremely (n=8), I don’t want to answer this question (n=116)

…advantageous?”

1-not at all (n=21), 2 (n=40), 3 (n=38), 4 (n=91), 5 (n=96), 6 (n=78), 7-extremely (n=93), I don’t want to answer this question (n=111)

…disadvantageous?”

1-not at all (n=176), 2 (n=76), 3 (n=68), 4 (n=75), 5 (n=31), 6 (n=16), 7-extremely (n=8), I don’t want to answer this question (n=118)

…beneficial?”

1-not at all (n=18), 2 (n=39), 3 (n=37), 4 (n=91), 5 (n=99), 6 (n=80), 7-extremely (n=95), I don’t want to answer this question (n=109)

…harmful?”

1-not at all (n=185), 2 (n=75), 3 (n=63), 4 (n=79), 5 (n=30), 6 (n=13), 7-extremely (n=6), I don’t want to answer this question (n=117)

…necessary?”

1-not at all (n=39), 2 (n=47), 3 (n=50), 4 (n=95), 5 (n=81), 6 (n=61), 7-extremely (n=84), I don't want to answer this question (n=111)

…unnecessary?"

1-not at all (n=172), 2 (n=55), 3 (n=63), 4 (n=84), 5 (n=34), 6 (n=26), 7-extremely (n=13), I don't want to answer this question (n=118)

Q21: "For the public society, to what extent do you think that Pre-registrations are/can be...

be...

Type for each subquestion: single choice

…useful?"

1-not at all (n=44), 2 (n=43), 3 (n=49), 4 (n=94), 5 (n=88), 6 (n=68), 7-extremely (n=67), I don't want to answer this question (n=115)

…useless?"

1-not at all (n=151), 2 (n=67), 3 (n=64), 4 (n=93), 5 (n=30), 6 (n=17), 7-extremely (n=25), I don't want to answer this question (n=121)

…advantageous?"

1-not at all (n=36), 2 (n=42), 3 (n=52), 4 (n=110), 5 (n=81), 6 (n=63), 7-extremely (n=66), I don't want to answer this question (n=118)

…disadvantageous?"

1-not at all (n=189), 2 (n=66), 3 (n=62), 4 (n=90), 5 (n=17), 6 (n=10), 7-extremely (n=12), I don't want to answer this question (n=122)

…beneficial?”

1-not at all (n=34), 2 (n=45), 3 (n=43), 4 (n=112), 5 (n=81), 6 (n=63), 7-extremely (n=69), I don’t want to answer this question (n=121)

…harmful?”

1-not at all (n=218), 2 (n=53), 3 (n=52), 4 (n=77), 5 (n=17), 6 (n=13), 7-extremely (n=8), I don’t want to answer this question (n=125)

…necessary?”

1-not at all (n=54), 2 (n=55), 3 (n=61), 4 (n=99), 5 (n=63), 6 (n=56), 7-extremely (n=60), I don’t want to answer this question (n=120)

…unnecessary?”

1-not at all (n=151), 2 (n=53), 3 (n=54), 4 (n=97), 5 (n=33), 6 (n=24), 7-extremely (n=32), I don’t want to answer this question (n=124)

Q22: “In the last 12 months, have you pre-registered any of your research projects?”

Type: single choice

Answer options:

Yes (n=37)

No (n=476)

I don’t want to answer this question (n=55)

Q23: “In the following 12 months, are you planning to pre-register any of your future research projects?”

Type: single choice

Answer options:

Yes (n=68)

No (n=386)

I don’t want to answer this question (n=114)

Q24: “If you feel that the previous questions did not completely capture your opinion about Pre-registrations, you can use the following blank for developing your thoughts:”

Type: free text

The comments are not reported for reasons of brevity and anonymity of the respondents.

Registered Reports

Definition: Publication format consisting of two peer-reviewed stages: 1) An in-principle acceptance of a research paper prior to data collection based on a detailed preregistration, and 2) The actual publication of the research paper after data collection, including any result (statistically significant or not) obtained by following what was specified in the preregistration.

Q25: "To what extent are you knowledgeable about Registered Reports?"

Type: single choice

Answer options:

1-not at all (n=276), 2 (n=82), 3 (n=64), 4 (n=64), 5 (n=43), 6 (n=20), 7-extremely (n=5), I don't want to answer this question (n=14)

Q26: "To what extent would you like to know more about Registered Reports?"

Type: single choice

Answer options:

1-not at all (n=36), 2 (n=37), 3 (n=53), 4 (n=76), 5 (n=102), 6 (n=118), 7-extremely (n=130), I don't want to answer this question (n=16)

Q27: "For your daily life as a researcher, to what extent do you think that Registered Reports are/can be...

be...

Type for each subquestion: single choice

…useful?"

1-not at all (n=36), 2 (n=31), 3 (n=40), 4 (n=79), 5 (n=93), 6 (n=78), 7-extremely (n=98), I don't want to answer this question (n=113)

…useless?”

1-not at all (n=181), 2 (n=38), 3 (n=52), 4 (n=62), 5 (n=28), 6 (n=24), 7-extremely (n=14), I don’t want to answer this question (n=124)

…advantageous?”

1-not at all (n=30), 2 (n=35), 3 (n=41), 4 (n=74), 5 (n=96), 6 (n=83), 7-extremely (n=91), I don’t want to answer this question (n=118)

…disadvantageous?”

1-not at all (n=172), 2 (n=95), 3 (n=52), 4 (n=60), 5 (n=40), 6 (n=14), 7-extremely (n=11), I don’t want to answer this question (n=124)

…beneficial?”

1-not at all (n=32), 2 (n=33), 3 (n=39), 4 (n=87), 5 (n=87), 6 (n=79), 7-extremely (n=95), I don’t want to answer this question (n=116)

…harmful?”

1-not at all (n=193), 2 (n=87), 3 (n=59), 4 (n=58), 5 (n=28), 6 (n=10), 7-extremely (n=9), I don’t want to answer this question (n=124)

…necessary?”

1-not at all (n=49), 2 (n=43), 3 (n=46), 4 (n=110), 5 (n=67), 6 (n=58), 7-extremely (n=76), I don’t want to answer this question (n=119)

…unnecessary?”

1-not at all (n=159), 2 (n=56), 3 (n=63), 4 (n=82), 5 (n=34), 6 (n=29), 7-extremely (n=20), I don’t want to answer this question (n=125)

Q28: “For your research field, to what extent do you think that Registered Reports are/can be...

be...

Type for each subquestion: single choice

…useful?”

1-not at all (n=33), 2 (n=28), 3 (n=45), 4 (n=70), 5 (n=71), 6 (n=83), 7-extremely (n=120), I don’t want to answer this question (n=118)

…useless?”

1-not at all (n=189), 2 (n=76), 3 (n=54), 4 (n=57), 5 (n=32), 6 (n=17), 7-extremely (n=17), I don’t want to answer this question (n=126)

…advantageous?”

1-not at all (n=34), 2 (n=29), 3 (n=36), 4 (n=78), 5 (n=84), 6 (n=77), 7-extremely (n=109), I don’t want to answer this question (n=121)

…disadvantageous?”

1-not at all (n=177), 2 (n=91), 3 (n=50), 4 (n=67), 5 (n=33), 6 (n=14), 7-extremely (n=10), I don’t want to answer this question (n=126)

…beneficial?”

1-not at all (n=30), 2 (n=28), 3 (n=43), 4 (n=83), 5 (n=75), 6 (n=79), 7-extremely (n=111), I don’t want to answer this question (n=119)

…harmful?”

1-not at all (n=193), 2 (n=79), 3 (n=62), 4 (n=59), 5 (n=27), 6 (n=12), 7-extremely (n=10), I don’t want to answer this question (n=126)

…necessary?”

1-not at all (n=48), 2 (n=45), 3 (n=42), 4 (n=96), 5 (n=68), 6 (n=54), 7-extremely (n=94), I don't want to answer this question (n=121)

…unnecessary?"

1-not at all (n=160), 2 (n=70), 3 (n=55), 4 (n=74), 5 (n=37), 6 (n=27), 7-extremely (n=19), I don't want to answer this question (n=126)

Q29: "For the public society, to what extent do you think that Registered Reports are/can be...

Type for each subquestion: single choice

…useful?"

1-not at all (n=41), 2 (n=43), 3 (n=40), 4 (n=99), 5 (n=85), 6 (n=61), 7-extremely (n=67), I don't want to answer this question (n=130)

…useless?"

1-not at all (n=148), 2 (n=83), 3 (n=56), 4 (n=78), 5 (n=21), 6 (n=21), 7-extremely (n=24), I don't want to answer this question (n=137)

…advantageous?"

1-not at all (n=36), 2 (n=48), 3 (n=39), 4 (n=107), 5 (n=84), 6 (n=55), 7-extremely (n=65), I don't want to answer this question (n=134)

…disadvantageous?"

1-not at all (n=188), 2 (n=80), 3 (n=48), 4 (n=75), 5 (n=13), 6 (n=13), 7-extremely (n=11), I don't want to answer this question (n=140)

…beneficial?"

1-not at all (n=36), 2 (n=42), 3 (n=45), 4 (n=110), 5 (n=77), 6 (n=64), 7-extremely (n=63), I don't want to answer this question (n=131)

…harmful?"

1-not at all (n=205), 2 (n=77), 3 (n=43), 4 (n=68), 5 (n=17), 6 (n=9), 7-extremely (n=9), I don't want to answer this question (n=140)

…necessary?"

1-not at all (n=55), 2 (n=47), 3 (n=56), 4 (n=116), 5 (n=50), 6 (n=50), 7-extremely (n=60), I don't want to answer this question (n=134)

…unnecessary?"

1-not at all (n=139), 2 (n=72), 3 (n=50), 4 (n=90), 5 (n=24), 6 (n=31), 7-extremely (n=23), I don't want to answer this question (n=139)

Q30: "In the last 12 months, have you published any paper as a Registered Report?"

Type: single choice

Answer options:

Yes (n=10)

No (n=502)

I don't want to answer this question (n=56)

Q31: "In the following 12 months, are you planning to publish any paper as a Registered Report?"

Type: single choice

Answer options:

Yes (n=33)

No (n=424)

I don’t want to answer this question (n=111)

Q32: “If you feel that the previous questions did not completely capture your opinion about Registered Reports you can use the following blank for developing your thoughts:”

Type: free text

The comments are not reported for reasons of brevity and anonymity of the respondents.

Replication Studies

Definition: Studies intended to 1) reproduce a scientific finding from a previous study by recreating the critical elements that are assumed to explain the original result (i.e., close replication), or 2) generalize a scientific finding by purposefully modifying at least one component of the original study, such as the sample, the protocol, or the material used (i.e., conceptual replication).

Q33: "To what extent are you knowledgeable about Replication Studies?"

Type: single choice

Answer options:

1-not at all (n=80), 2 (n=67), 3 (n=74), 4 (n=95), 5 (n=123), 6 (n=81), 7-extremely (n=37), I don't want to answer this question (n=11)

Q34: "To what extent would you like to know more about Replication Studies?"

Type: single choice

Answer options:

1-not at all (n=29), 2 (n=40), 3 (n=52), 4 (n=87), 5 (n=125), 6 (n=99), 7-extremely (n=125), I don't want to answer this question (n=11)

Q35: "For your daily life as a researcher, to what extent do you think that Replication Studies are/can be...

Type for each subquestion: single choice

…useful?"

1-not at all (n=12), 2 (n=11), 3 (n=28), 4 (n=60), 5 (n=83), 6 (n=128), 7-extremely (n=201), I don't want to answer this question (n=45)

…useless?"

1-not at all (n=304), 2 (n=104), 3 (n=47), 4 (n=35), 5 (n=15), 6 (n=5), 7-extremely (n=5), I don’t want to answer this question (n=53)

…advantageous?”

1-not at all (n=10), 2 (n=18), 3 (n=26), 4 (n=78), 5 (n=108), 6 (n=110), 7-extremely (n=171), I don’t want to answer this question (n=47)

…disadvantageous?”

1-not at all (n=295), 2 (n=100), 3 (n=60), 4 (n=42), 5 (n=12), 6 (n=4), 7-extremely (n=1), I don’t want to answer this question (n=54)

…beneficial?”

1-not at all (n=8), 2 (n=16), 3 (n=21), 4 (n=71), 5 (n=96), 6 (n=125), 7-extremely (n=184), I don’t want to answer this question (n=47)

…harmful?”

1-not at all (n=314), 2 (n=99), 3 (n=51), 4 (n=32), 5 (n=11), 6 (n=4), 7-extremely (n=7), I don’t want to answer this question (n=55)

…necessary?”

1-not at all (n=15), 2 (n=17), 3 (n=27), 4 (n=76), 5 (n=81), 6 (n=107), 7-extremely (n=197), I don’t want to answer this question (n=48)

…unnecessary?”

1-not at all (n=304), 2 (n=88), 3 (n=57), 4 (n=47), 5 (n=5), 6 (n=4), 7-extremely (n=5), I don’t want to answer this question (n=55)

Q36: “For your research field, to what extent do you think that Replication Studies are/can be...

Type for each subquestion: single choice

…useful?”

1-not at all (n=11), 2 (n=14), 3 (n=11), 4 (n=51), 5 (n=76), 6 (n=116), 7-extremely (n=247), I don’t want to answer this question (n=42)

…useless?”

1-not at all (n=323), 2 (n=98), 3 (n=51), 4 (n=26), 5 (n=9), 6 (n=8), 7-extremely (n=3), I don’t want to answer this question (n=50)

…advantageous?”

1-not at all (n=9), 2 (n=19), 3 (n=16), 4 (n=61), 5 (n=87), 6 (n=123), 7-extremely (n=207), I don’t want to answer this question (n=46)

…disadvantageous?”

1-not at all (n=316), 2 (n=96), 3 (n=55), 4 (n=34), 5 (n=11), 6 (n=3), 7-extremely (n=1), I don’t want to answer this question (n=52)

…beneficial?”

1-not at all (n=5), 2 (n=17), 3 (n=19), 4 (n=58), 5 (n=84), 6 (n=127), 7-extremely (n=213), I don’t want to answer this question (n=45)

…harmful?”

1-not at all (n=338), 2 (n=92), 3 (n=48), 4 (n=27), 5 (n=8), 6 (n=4), 7-extremely (n=0), I don’t want to answer this question (n=51)

…necessary?”

1-not at all (n=12), 2 (n=19), 3 (n=23), 4 (n=66), 5 (n=67), 6 (n=116), 7-extremely (n=221), I don’t want to answer this question (n=44)

…unnecessary?”

1-not at all (n=321), 2 (n=94), 3 (n=44), 4 (n=41), 5 (n=7), 6 (n=5), 7-extremely (n=4), I don’t want to answer this question (n=52)

Q37: “For the public society, to what extent do you think that Replication Studies are/can be...

Type for each subquestion: single choice

…useful?”

1-not at all (n=25), 2 (n=16), 3 (n=18), 4 (n=59), 5 (n=74), 6 (n=104), 7-extremely (n=213), I don’t want to answer this question (n=59)

…useless?”

1-not at all (n=303), 2 (n=88), 3 (n=48), 4 (n=39), 5 (n=11), 6 (n=6), 7-extremely (n=9), I don’t want to answer this question (n=64)

…advantageous?”

1-not at all (n=23), 2 (n=17), 3 (n=24), 4 (n=70), 5 (n=71), 6 (n=110), 7-extremely (n=188), I don’t want to answer this question (n=65)

…disadvantageous?”

1-not at all (n=320), 2 (n=78), 3 (n=51), 4 (n=38), 5 (n=4), 6 (n=3), 7-extremely (n=6), I don’t want to answer this question (n=68)

…beneficial?”

1-not at all (n=24), 2 (n=17), 3 (n=20), 4 (n=72), 5 (n=73), 6 (n=101), 7-extremely (n=199), I don’t want to answer this question (n=62)

…harmful?”

1-not at all (n=341), 2 (n=69), 3 (n=42), 4 (n=34), 5 (n=7), 6 (n=3), 7-extremely (n=5), I don’t want to answer this question (n=67)

…necessary?”

1-not at all (n=29), 2 (n=20), 3 (n=27), 4 (n=64), 5 (n=67), 6 (n=99), 7-extremely (n=199), I don’t want to answer this question (n=63)

…unnecessary?”

1-not at all (n=297), 2 (n=84), 3 (n=53), 4 (n=43), 5 (n=8), 6 (n=6), 7-extremely (n=10), I don’t want to answer this question (n=67)

Q38: “In the last 12 months, have you conducted or collaborated in any Replication Study?”

Type: single choice

Answer options:

Yes (n=68)

No (n=467)

I don’t want to answer this question (n=33)

Q39: “In the following 12 months, are you planning to conduct or collaborate in any Replication Study?”

Type: single choice

Answer options:

Yes (n=66)

No (n=434)

I don’t want to answer this question (n=68)

Q40: “If you feel that the previous questions did not completely capture your opinion about Replication Studies you can use the following blank for developing your thoughts:”

Type: free text

The comments are not reported for reasons of brevity and anonymity of the respondents.

Section 2 - Local Support

This section intended to analyze how researchers at the Max Planck Society are locally supported in matters related to Open Science. It contained 7 questions and 4 conditional questions which are not reported in this research article.

Section 3 - General Information and Demographics

Q48: "How old are you?"

Type: single choice

Answer option:

0 (n=1), 9 (n=1), 22 (n=2), 23 (n=6), 24 (n=30), 25 (n=36), 26 (n=75), 27 (n=107), 28 (n=75), 29 (n=66), 30 (n=47), 31 (n=27), 32 (n=19), 33 (n=16), 34 (n=4), 35 (n=6), 37 (n=2), 38 (n=1), 39 (n=1), 40 (n=2), 42 (n=1), 43 (n=1), 44 (n=1), I don't want to answer this question (n=44)

Q49: "What was your sex assigned at birth?"

Type: single choice

Answer options:

Female (n=249)

Male (n=286)

Intersex (n=0)

Other (n=1)

I don't want to answer this question (n=32)

Q50: "Which Max Planck Institute are you associated with?"

Type: single choice

The results of this question are not analysed in the research article to guarantee the anonymity of the respondents.

Q51: “Which scientific section does the institute you are associated with belongs to?”

Type: single choice

Answer options:

Biology and Medicine (BM) section (n=205), Chemistry, Physics and Technology (CPT) section (n=247), Human Sciences (HUM) section (n=92), I don’t want to answer this question (n=25)
